# FTO downregulation mediated by hypoxia facilitates colorectal cancer metastasis

**DOI:** 10.1038/s41388-021-01916-0

**Published:** 2021-07-03

**Authors:** Dan-Yun Ruan, Ting Li, Ying-Nan Wang, Qi Meng, Yang Li, Kai Yu, Min Wang, Jin-Fei Lin, Li-Zhi Luo, De-Shen Wang, Jun-Zhong Lin, Long Bai, Ze-Xian Liu, Qi Zhao, Xiang-Yuan Wu, Huai-Qiang Ju, Rui-Hua Xu

**Affiliations:** 1 State Key Laboratory of Oncology in South China, Collaborative Innovation Center for Cancer Medicine, Sun Yat-sen University Cancer Center, Guangzhou, P. R. China; 2 Department of Guangdong Key Laboratory of Liver Disease, The Third Affiliated Hospital of Sun Yat-sen University, Guangzhou, China; 3 Department of Medical Oncology, Sun Yat-sen University Cancer Center, Guangzhou, P. R. China; 4 Department of Colorectal Surgery, Sun Yat-sen University Cancer Center, Guangzhou, P. R. China; 5 Research Unit of Precision Diagnosis and Treatment for Gastrointestinal Cancer, Chinese Academy of Medical Sciences, Guangzhou, China

**Keywords:** Colorectal cancer, Epigenetics

## Abstract

Fat mass and obesity-associated protein (FTO), an N6-methyladenosine (m^6^A) demethylase, participates in tumor progression and metastasis in many malignancies, but its role in colorectal cancer (CRC) is still unclear. Here, we found that FTO protein levels, but not RNA levels, were downregulated in CRC tissues. Reduced FTO protein expression was correlated with a high recurrence rate and poor prognosis in resectable CRC patients. Moreover, we demonstrated that hypoxia restrained FTO protein expression, mainly due to an increase in ubiquitin-mediated protein degradation. The serine/threonine kinase receptor associated protein (STRAP) might served as the E3 ligase and K216 was the major ubiquitination site responsible for hypoxia-induced FTO degradation. FTO inhibited CRC metastasis both in vitro and in vivo. Mechanistically, FTO exerted a tumor suppressive role by inhibiting metastasis-associated protein 1 (MTA1) expression in an m^6^A-dependent manner. Methylated *MTA1* transcripts were recognized by an m^6^A “reader”, insulin-like growth factor 2 mRNA binding protein 2 (IGF2BP2), which then stabilized its mRNA. Together, our findings highlight the critical role of FTO in CRC metastasis and reveal a novel epigenetic mechanism by which the hypoxic tumor microenvironment promotes CRC metastasis.

## Introduction

Colorectal cancer (CRC) is the most common gastrointestinal malignancy and the second leading cause of cancer death worldwide [1]. Increasing incidence and mortality make CRC a major public health problem, and the high mortality rate is mostly due to recurrence and metastasis [2, 3]. Tumor metastasis is a dynamic and complex progressive process affected by many factors. Recently, N6-methyladenosine (m^6^A), as the most prevalent mRNA modification, has been noted to play an important role in tumor metastasis in various types of cancers [4–6]. Therefore, exploring the regulation of RNA m^6^A modification in CRC recurrence and metastasis is of great significance for improving CRC patient prognosis.

M^6^A modification is involved in cancer pathogenesis and progression through the m^6^A enzyme system, which is mainly composed of methyltransferases (“writers”), demethylases (“erasers”) and binding proteins (“readers”) [5, 7]. These m^6^A enzymes participate in the development of cancers, such as gliomas, leukemia, and breast cancer, by affecting different stages of the RNA life cycle, including pre-mRNA splicing, export, translation, and stability [8–12]. Recently, we first identified the function of m^6^A writer methyltransferase like 3 (METTL3) in promoting CRC progression by maintaining the CRC cell stemness phenotype via an m^6^A-dependent mechanism [13]. Meanwhile, other studies have reported that m^6^A writers facilitate CRC development in an m^6^A-dependent manner [14, 15]. There are two known m^6^A-specific erasers, AlkB homolog 5 (ALKBH5) and fat mass- and obesity-associated protein (FTO). FTO was first identified as a gene related to obesity and energy metabolism and was then identified as the RNA m^6^A demethylase [16]. The role of FTO in tumor development and prognosis is inconsistent among different types of cancers. FTO was found to play oncogenic roles in acute myeloid leukemia, melanoma, breast cancer, and cervical cancer through its posttranscriptional regulation function [6, 9, 17, 18]. Meanwhile, several studies have reported that FTO has tumor suppressor activity and is inversely correlated with tumor progression in ovarian cancer, hepatocellular carcinoma, intrahepatic cholangiocarcinoma and renal cell carcinoma [19–22]. However, the role of FTO in CRC as an m^6^A demethylase remains poorly understood.

In this study, we first demonstrated that FTO expression was downregulated in CRC tumor tissue and that higher FTO expression was associated with better prognosis in CRC patients. Then, we revealed that FTO inhibited tumor metastasis and progression in vitro and in vivo by decreasing the expression of its downstream target gene, metastasis-associated protein 1 (MTA1). FTO precisely reduced the m^6^A level of *MTA1* transcripts, resulting in decreased mRNA stability. Moreover, we found hypoxia could induced FTO downregulation mainly through the ubiquitin-mediated protein degradation. We also found serine/threonine kinase receptor-associated protein STRAP) was probably the E3 ligase of FTO and Lysine(K)-216 was its major ubiquitination site. Overall, our findings revealed a critical role of FTO as a predictor of metastasis and recurrence of colorectal cancer and elucidated a new epigenetic mechanism through which the hypoxic tumor microenvironment promotes CRC metastasis.

## Results

### FTO downregulation is associated with poor clinical prognosis in CRC tissues

**Fig. 1:**
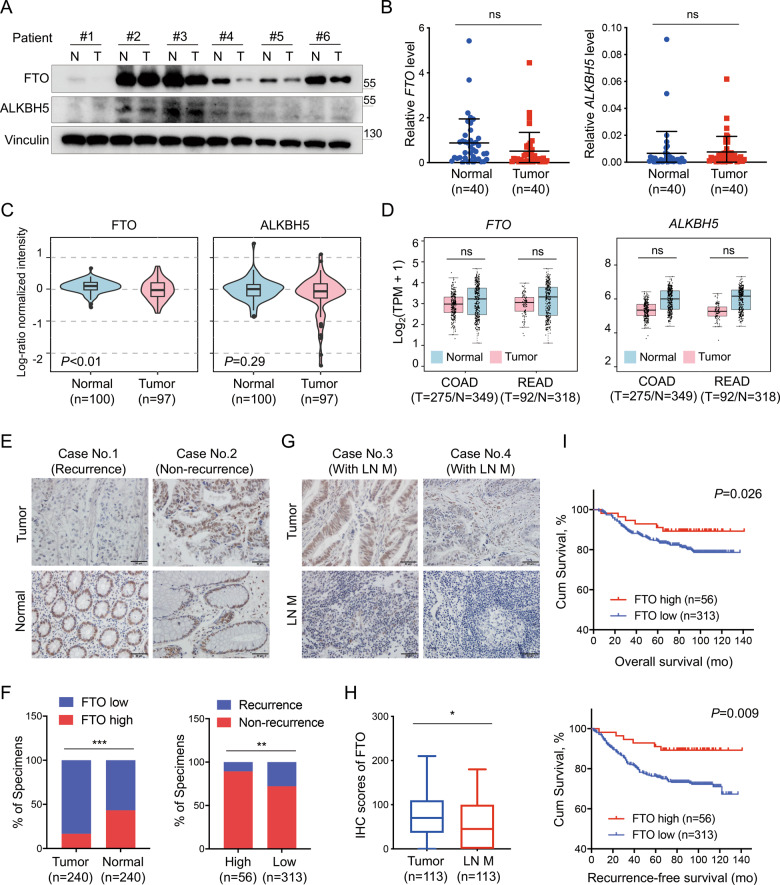
FTO is downregulated in CRC tissues, and its downregulation is associated with poor clinical prognosis. **A** FTO and ALKBH5 protein levels in six paired CRC tumor (T) and adjacent normal tissues (N) determined by immunoblotting assay. **B** Real-time PCR analysis of mRNA expression of FTO and ALKBH5 in 40 paired CRC tumor and adjacent normal tissues from SYSUCC. GAPDH was used as a control, and comparisons were analyzed by two-tailed paired Student’s *t* test. **C** Comparison of the proteogenomic expression of FTO and ALKBH5 in tumor and normal tissue from the CPTAC Data Portal of colon cancer (https://cptac-data-portal.georgetown.edu/cptac/s/S045). **D** Comparison of FTO and ALKBH5 expression in normal and cancer transcriptomes obtained from TCGA and GTEx datasets of COAD and READ, analyzed by the online tool GEPIA (http://gepia.cancer-pku.cn/index.html). **E**, **F** Representative immunohistochemistry (IHC) images and IHC staining scores of FTO expression in CRC tumor tissues and paired adjacent normal tissues from cases with or without recurrence (*n* = 240). FTO level was classified as “high” and “low” according to the median score (≤150). Scale bar: 50 μm. **G**, **H** Representative IHC images and comparison of FTO expression in paired CRC tumor tissue and lymph node metastasis (LN M) from 113 stage III CRC patients. Scale bar: 50 μm. **I** Kaplan–Meier analysis of overall survival (OS) and recurrence-free survival (RFS) in CRC patients with different FTO expression levels (*n* = 369). *P < 0.05, **P < 0.01, ***P < 0.001.

M^6^A modification is a dynamic process mediated by enzymes known as writers and erasers. We previously revealed the oncogenic role of the m^6^A writer METTL3 in CRC [13], and we speculated that erasers might also be involved in the progression of CRC. To investigate m^6^A eraser expression in CRC, we first analyzed FTO and ALKBH5 mRNA and protein levels in paired CRC tumor and adjacent normal tissues from Sun Yat-sen University Cancer Center (SYSUCC). We found that FTO was downregulated at the protein level but not at the mRNA level in CRC tumor tissues, while ALKBH5 expression did not show any differences (Fig. 1A, B, Supplementary Information: Fig. S1A). Then, we compared FTO and ALKBH5 transcriptome and proteogenomic expression between tumor and normal tissues in The Cancer Genome Atlas (TCGA), the Genotype-Tissue Expression (GTEx) dataset (http://gepia.cancer-pku.cn/index.html) and the Clinical Proteomic Tumor Analysis Consortium (CPTAC) Data Portal (https://cptac-data-portal.georgetown.edu/cptac/s/S045) [23]. As expected, only FTO showed lower protein expression in CRC tumor tissue (Fig. 1C, D). To verify the expression pattern of FTO, we performed an immunohistochemical (IHC) staining assay to analyze its protein expression level in 240 paired tumor and adjacent normal tissues from SYSUCC CRC patients (Fig. 1E). The clinicopathological characteristics were also collected for further analysis. Consistent with the above observations, FTO was significantly downregulated in CRC tumor tissues compared to normal tissues according to the IHC results, and patients with lower expression of FTO had a higher probability of tumor recurrence (Fig. 1F). Furthermore, we analyzed 113 stage III patients with paired tumor tissues and lymph node metastasis specimens and found that FTO expression was downregulated in lymph node metastasis tissue compared with the primary tumor (Fig. 1G, H). However, there were no significant correlations between clinicopathological features and tumor FTO expression (Supplementary Information: Table S1). We next performed survival analysis in 369 stage I–III CRC cases that received radical surgery and found that cases with low FTO expression exhibited both poorer overall survival (OS) and poorer recurrence-free survival (RFS) (Fig. 1I). Multivariate analysis showed that FTO served as an independent predictive factor of OS and RFS (Supplementary Information: Table S2). These results suggest that the m^6^A eraser FTO is downregulated in CRC and is a potential prognostic indicator in CRC patients.

### FTO suppresses CRC cell migration and invasion in vitro

**Fig. 2:**
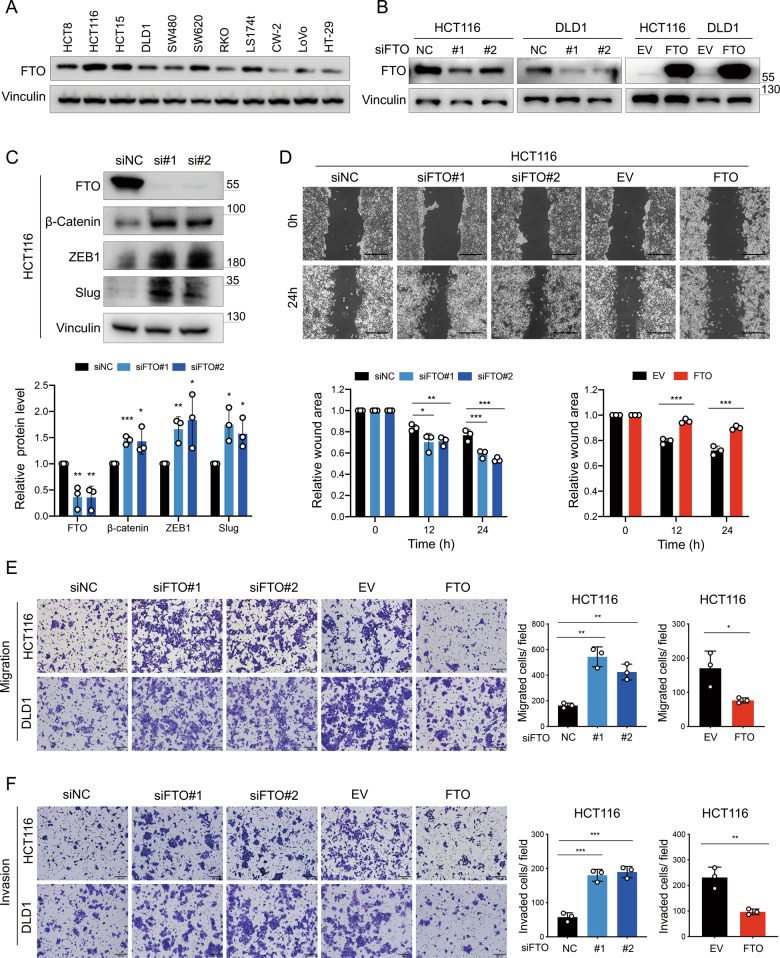
FTO suppresses CRC cell migration and invasion in vitro. **A** Immunoblotting of FTO expression in human colorectal cancer cells (HCT8, HCT116, HCT15, DLD1, SW480, SW620, RKO, Ls174t, CW-2, LoVo, and HT-29). **B** Immunoblotting of normal control (NC) versus FTO knockdown with si#1, si#2 and empty vector (EV) versus FTO-overexpressing (FTO) efficiencies in HCT116 and DLD1 cells. **C** Immunoblotting (up) and bar graph (down) of EMT markers (β-Catenin, ZEB1, Slug) in HCT116 and DLD1 cells after FTO knockdown. **D** Wound healing assays of FTO knockdown and FTO overexpression HCT116 cells were recorded (up) and quantitatively analyzed (down). Scale bar: 400 μm. Two-way ANOVA was used for comparisons at each time point. **E** Images and quantification of transwell migration assays of FTO knockdown and FTO overexpression HCT116 and DLD1 cells. Scale bar: 200 μm. **F** Images and quantification of invaded FTO knockdown and FTO overexpression HCT116 and DLD1 cells. Scale bar: 200 μm. Data in (**C**, **D**, **E**, and **F**) are presented as the means ± S.D. (*n* = 3) *P < 0.05, **P < 0.01, ***P < 0.001 (Student’s *t* test).

To investigate the relationship between FTO and the metastasis-related phenotype, we first detected the protein level of FTO in 11 colorectal cancer cell lines (HCT8, HCT116, HCT15, DLD1, RKO, SW480, SW620, Ls174t, CW-2, LoVo, and HT-29) and chose two cell lines with different FTO levels, HCT116 and DLD1, for further experiments (Fig. 2A, Supplementary Information: Fig. S2A). We next knocked down FTO and constructed stable FTO-overexpressing HCT116 and DLD1 cell lines (Fig. 2B). FTO knockdown increased the expression of epithelial–mesenchymal transition (EMT) related markers (β-catenin, ZEB1 and Slug) (Figs. 2C, S2B). As determined by wound healing assays and cell migration assays, FTO deficiency enhanced cell migration, while FTO overexpression inhibited cell migration, in CRC cells (Figs. 2D, E, S2C–F). Meanwhile, cell invasion assays revealed that cell invasion ability was negatively correlated with the expression of FTO (Figs. 2F, S2G). In addition, MTS assays showed that FTO knockdown accelerated cell proliferation, while FTO overexpression significantly inhibited cell growth (Fig. S2H).

### FTO inhibits CRC cell growth and metastasis in vivo

**Fig. 3:**
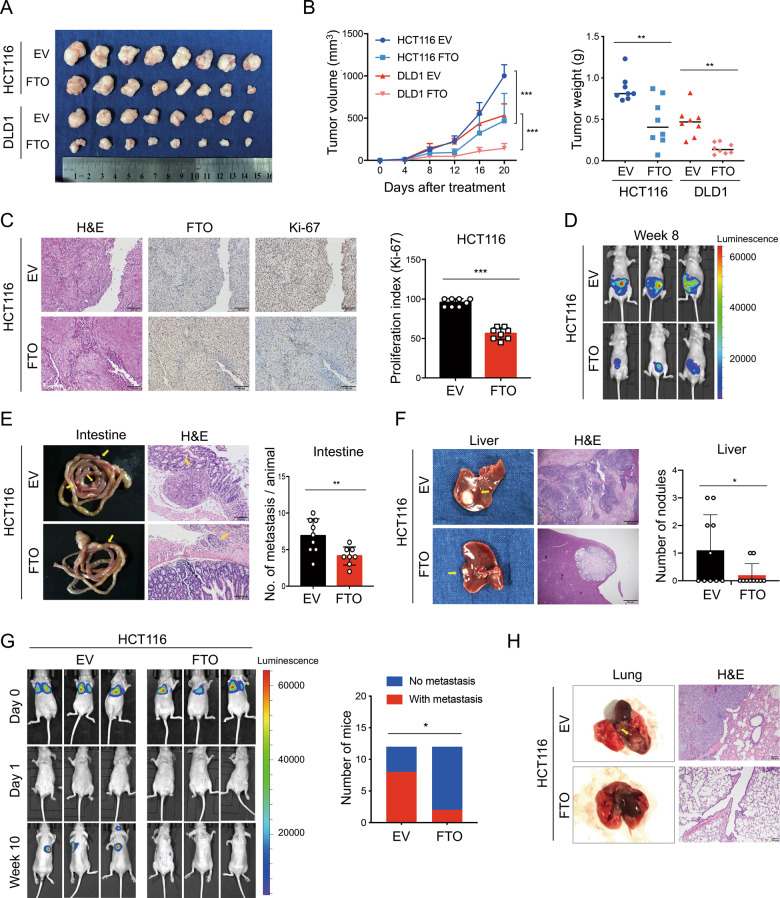
FTO inhibits CRC cell metastasis in vivo. **A**, **B** Photograph and quantification of excised subcutaneous tumors by implanting FTO-overexpressing (FTO) versus control empty vector (EV) HCT116 and DLD1 cells (*n* = 8 mice per group). Two-way ANOVA and *t* test analysis were applied for comparisons of tumor growth rate (left) and tumor weight (right), respectively. **C** Representative images of hematoxylin and eosin (H&E) staining and IHC staining of FTO and Ki-67 antibodies in paraffin-embedded mouse subcutaneous tumor sections derived from the HCT116 FTO and EV groups. Scale bar: 100 μm (left). The Ki-67 proliferation index (percent Ki-67-positive) was quantified in tumor sections from the FTO and EV groups (*n* = 8 mice per group), and the two-tailed Student’s *t* test was used for comparisons (right). **D** The bioluminescent imaging of nude mice orthotopic tumor model with FTO and EV luciferase-labeled HCT116 cells (*n* = 10 mice per group). **E** Representative images of intestinal metastatic nodules and H&E staining in FTO and EV groups were shown (left) and quantification were analyzed by Student’s *t* test (right). **F** Representative specimens and H&E staining photographs of liver metastatic nodules were shown (left) and quantification were analyzed by Student’s *t* test (right). **G** The bioluminescent imaging of nude mice tail vein injection metastasis model with FTO and EV luciferase-labeled HCT116 cells at days zero, one and week ten (*n* = 12 mice per group). Tumor metastasis formation were followed at week ten and distant metastasis in FTO and EV groups were analyzed by Pearson’s chi-square test. **H** Representative specimen and H&E staining photographs of the metastatic nodules in the lung. *P < 0.05, **P < 0.01, ***P < 0.001.

To evaluate the function of FTO in vivo, we applied a nude mouse subcutaneous xenograft model. CRC cell lines with FTO overexpression had a significantly lower tumor growth rate and tumor weight (Fig. 3A, B). Histologic examination of Ki-67 showed that tumors generated from FTO-overexpressing HCT116 and DLD1 cells had reduced cell proliferation indices (Fig. 3C, Supplementary Information: Fig. S3A). We further investigated the effect of FTO expression on tumor metastasis in vivo through two metastasis models with luciferase-labeled HCT116 cells. In the orthotopic tumor model, FTO overexpression reduced intestinal mesenteric metastasis and liver metastasis in mice bearing HCT116 cells (Fig. 3D, F). Similar results were observed in the tail vein metastasis model; FTO overexpression inhibited distant metastasis while FTO knockdown increased distant metastasis in mice injected with HCT116 cells (Figs. 3G, H, S3B–D). Taken together, our data indicated that FTO overexpression suppressed CRC growth and metastatic potential in vivo.

### MTA1 is a downstream target gene of FTO

**Fig. 4:**
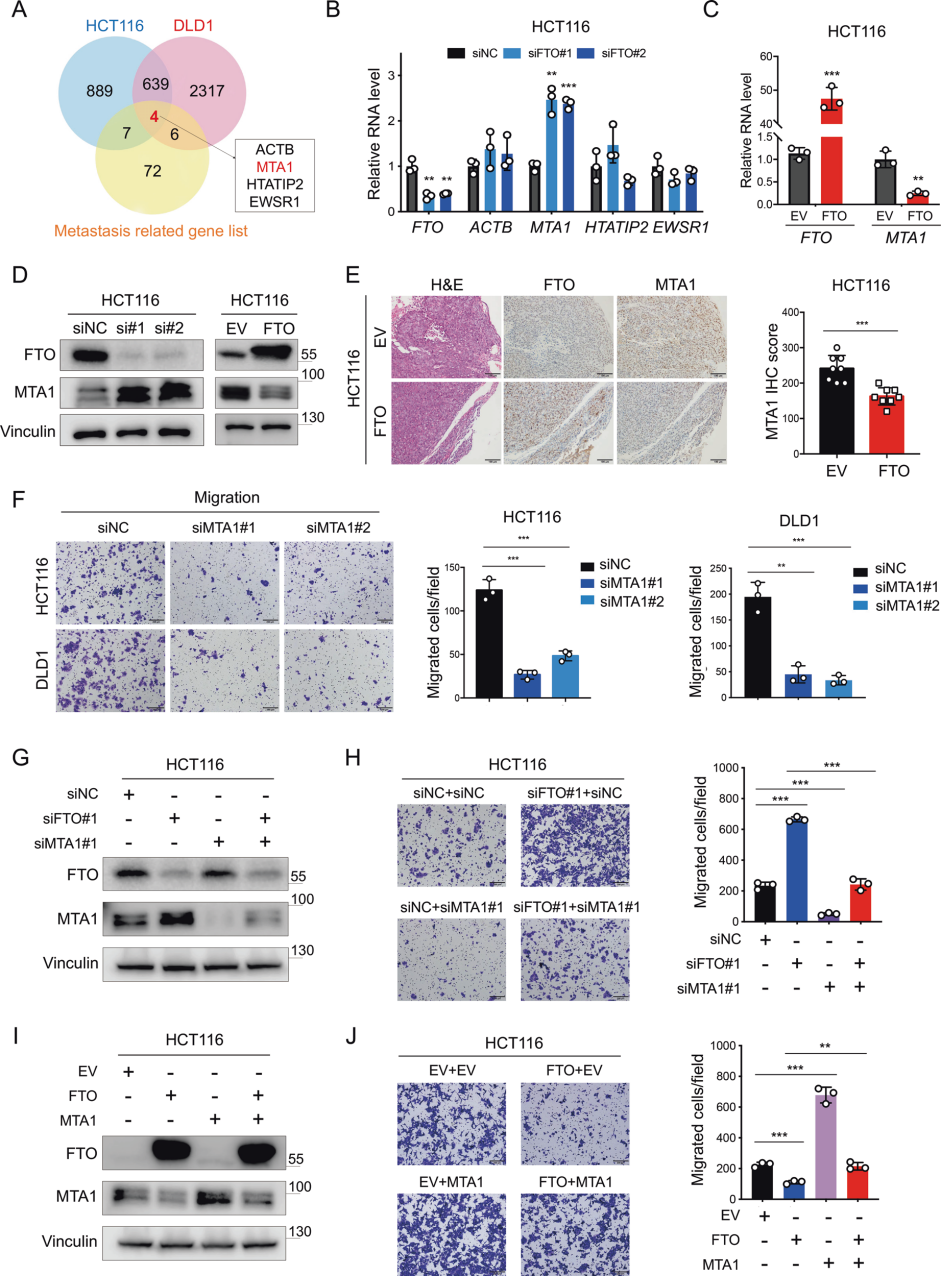
MTA1 is a downstream target gene of FTO. **A** Differentially expressed genes between FTO knockdown and normal control HCT116 and DLD1 cells (left) were identified by transcriptome sequencing. Venn diagram showing the overlap of significantly upregulated genes in FTO knockdown cells with a list of 84 human tumor metastasis-related genes [24, 25] and four downstream genes were screened. **B** RT-qPCR arrays were used to detect the RNA expression levels of *FTO*, *ACTB*, *MTA1*, *HTATIP2*, and *EWSR1* after FTO knockdown in HCT116 cells, normalized to *GAPDH*. **C** *MTA1* RNA expression levels were analyzed in FTO and EV HCT116 cells. **D** Immunoblot assay of MTA1 protein levels in HCT116 cells upon FTO knockdown and FTO overexpression. **E** Representative IHC images of H&E staining, FTO expression and MTA1 expression in FTO versus EV HCT116 induced tumor tissues (left). Scale bar: 100 μm. IHC scores of MTA1 expression in FTO versus EV group (*n* = 8 mice per group) compared by two-tailed Student’s *t* test (right). **F** The images (left) and quantification (right) of transwell migration assays of MTA1 knockdown (siMTA1#1, siMTA1#2) versus normal control (siNC) cells. Scale bar: 200 μm. **G** Immunoblot assay of FTO and MTA1 protein levels in siNC, siFTO#1, siMTA1#1, and siFTO#1 + siMTA1#1 HCT116 cells. **H** Images (left) and quantification (right) of migrated HCT116 cells with siNC+siNC, siFTO#1 + siNC, siNC + siMTA1#1, and siFTO#1 + siMTA1#1. **I** Immunoblotting assay of FTO and MTA1 protein levels in HCT116 cells with EV, FTO, MTA1 overexpression (MTA1) and FTO + MTA1. **J** The images (left) and quantification (right) of migrated HCT116 cells with EV + EV, FTO + EV, EV + MTA1 and FTO + MTA1. Scale bar: 200 μm. Data in (**B**, **C**, **F**, **H**, and **J**) are presented as the means ± S.D. (*n* = 3) *P < 0.05, **P < 0.01, ***P < 0.001 (Student’s *t* test).

To identify the underlying mechanisms by which FTO is involved in CRC progression and metastasis, we performed transcriptome sequencing analysis in HCT116 and DLD1 cells with FTO knockdown and normal controls. There were 1539 and 2966 genes significantly upregulated in FTO knockdown HCT116 and DLD1 cells, respectively (compared with the normal control). We next screened these genes for overlap with a list of 84 human tumor metastasis-related genes [24, 25] and identified four genes as potential downstream target genes of FTO (Fig. 4A). Among these four genes, only MTA1 exhibited significantly increased expression in FTO-depleted CRC cells (Fig. 4B, Supplementary Information: Fig. S4A). As expected, the *MTA1* level was decreased in FTO-overexpressing CRC cells (Figs. 4C, S4B). We next verified this result by examining the protein expression levels among the above CRC cells and confirmed that FTO expression attenuated MTA1 expression in CRC cell lines (Figs. 4D, S4C). Furthermore, histologic examination showed that FTO overexpression reduced MTA1 expression in tumor tissue (Figs. 4E, S4D).

We next explored whether MTA1 could reverse the effects of FTO depletion or overexpression on CRC cell migration and invasion. We first knocked down MTA1 in HCT116 and DLD1 cells and verified that MTA1 could accelerate CRC cell migration (Figs. 4F, S4E). Then, we constructed HCT116 and DLD1 cells with simultaneous FTO and MTA1 knockdown or overexpression. As expected, MTA1 knockdown notably impaired CRC cell invasion, and MTA1 deficiency in FTO-depleted CRC cells led to decreased CRC cell migration. Consistent with this, MTA1 overexpression significantly reversed the inhibition of CRC cell migration by FTO overexpression (Figs. 4G–J, S4F-4I). Together, these results suggested that MTA1 is the downstream target gene of FTO and that FTO inhibited CRC progression and metastasis through the regulation of MTA1 expression.

### MTA1 is regulated by FTO in an m^6^A-dependent manner

**Fig. 5:**
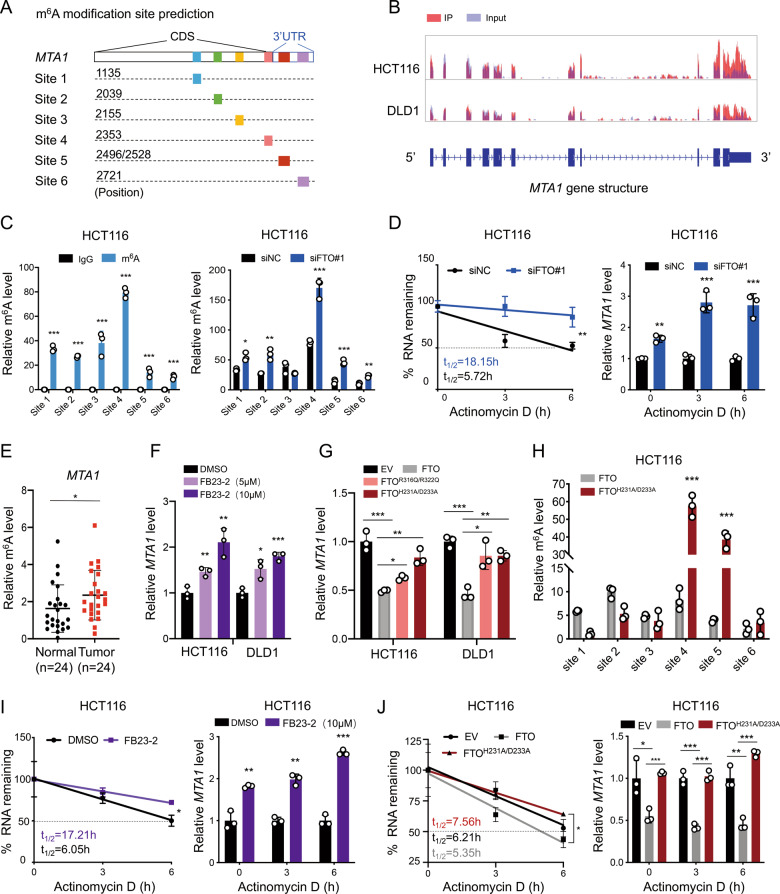
MTA1 is regulated by FTO in an m^6^A-dependent manner. **A** The possible m^6^A sites in *MTA1* transcripts. **B** M^6^A- RIP seq was performed in HCT116 and DLD1 cells, and the overlay track represents the m^6^A peaks with blue for input and red for IP. **C** Enrichment of m^6^A in different regions of *MTA1* mRNA was detected by m^6^A-RIP qPCR assay in HCT116 cells (left). The m^6^A methylation of *MTA1* mRNA regions in siFTO#1 versus siNC HCT116 cells by m^6^A MeRIP analysis (right). IgG was used as the negative control. The relative m^6^A enrichment was normalized to the input. The sequences are shown in Table S7. **D** The *MTA1* mRNA decay rate was analyzed by nonlinear regression (left), and the relative mRNA expression level of *MTA1* was compared between the two groups at each time point (right) in HCT116 cells. **E** Enrichment of m^6^A in site 4 of *MTA1* mRNA was detected by m^6^A-RIP qPCR assay in CRC tumor tissues versus paired adjacent normal tissues (*n* = 24). **F** Relative *MTA1* RNA expression levels in DMSO, FB23-2 (5 μM) and FB23-2 (10 μM) treated HCT116 and DLD1 cells. **G** Relative *MTA1* RNA expression levels in empty vector (EV), FTO overexpression wild type (FTO), FTO overexpression with R316Q/R322Q mutant (FTO^R316Q/R322Q^) and FTO overexpression with H231A/D233A mutant (FTO^H231A/D233A^) HCT116 and DLD1 cells. **H** Relative m^6^A level of *MTA1* mRNA regions in FTO versus FTO^H231A/D233A^ HCT116 cells by m^6^A MeRIP analysis. IgG was used as the negative control. Relative m^6^A level was normalized to the input. **I** The *MTA1* mRNA decay rate in DMSO versus FB23-2 (10 μM) treated HCT116 cells (left), and the relative mRNA expression level of *MTA1* was compared between the two groups at each time point (right). **J** The *MTA1* mRNA decay rate in EV, FTO, and FTO^H231A/D233A^ HCT116 cells (left), and the relative *MTA1* mRNA expression level in each group at the time point (right). Data in (**C**, **D**, **F**, **G**, **H**, **I**, and **J**) are presented as the means ± S.D. (*n* = 3) *P < 0.05, **P < 0.01, ***P < 0.001 (Student’s *t* test).

Recent studies have shown that FTO plays m^6^A-dependent roles in tumorigenesis and progression in several cancers. To elucidate the mechanism of FTO-mediated MTA1 regulation, we first predicted potential m^6^A modification sites on *MTA1* mRNA with SRAMP (http://www.cuilab.cn/sramp/) and labeled them as site 1 to site 6 regions. (Fig. 5A). Then, we assessed the m^6^A modification of *MTA1* mRNA by m^6^A pulldown sequencing in HCT116 and DLD1 cells, which indicated that most of the m^6^A peaks were located at the 3’ untranslated region (3’-UTR) and around the coding sequence (CDS)-3’UTR junction region (Fig. 5B). Further m^6^A pulldown validation showed that the site 4 region, which was located near the CDS-3’UTR junction region, was the most reliably modified region of *MTA1* transcripts. After FTO knockdown in CRC cells, m^6^A levels in the site 4 region were most significantly increased compared with normal control cells (Fig. 5C, Supplementary Information: Fig. S5A). In addition, after knocking down FTO, *MTA1* mRNA expression was initially increased, and the RNA decay rate was slower than that of the normal control in CRC cells (Figs. 5D, S5B). M^6^A enrichment in site 4 region of *MTA1* mRNA was also found increased in CRC tumor tissues versus paired adjacent normal tissues (Fig. 5E).

Moreover, we sought to confirm the role of FTO through its m^6^A demethylase function. Several FTO inhibitors were previously reported [26, 27], we treated CRC cells with 5 μM and 10 μM concentrations of FB23-2 for 24 h and the *MTA1* mRNA level was upregulated (Fig. 5F). We also constructed two double mutant FTO plasmids: the two α-KG ligands mutated R316Q/R322Q and the two iron(II) ligands mutated H231A/D233A, which as previously reported, completely aborted the m^6^A demethylation activity [16]. Similar results were observed in the FTO mutant groups (Fig. 5G) and m^6^A levels in FTO H231A/D233A mutant cells were significantly increased compared with FTO wild type cells (Fig. 5H). After FB23-2 treated, or in FTO H231A/D233A mutant HCT116 cells*, MTA1* mRNA decay rate was slower than that of the normal control group (Fig. 5I, J).

### IGF2BP2 specifically binds the *MTA1* transcripts

**Fig. 6:**
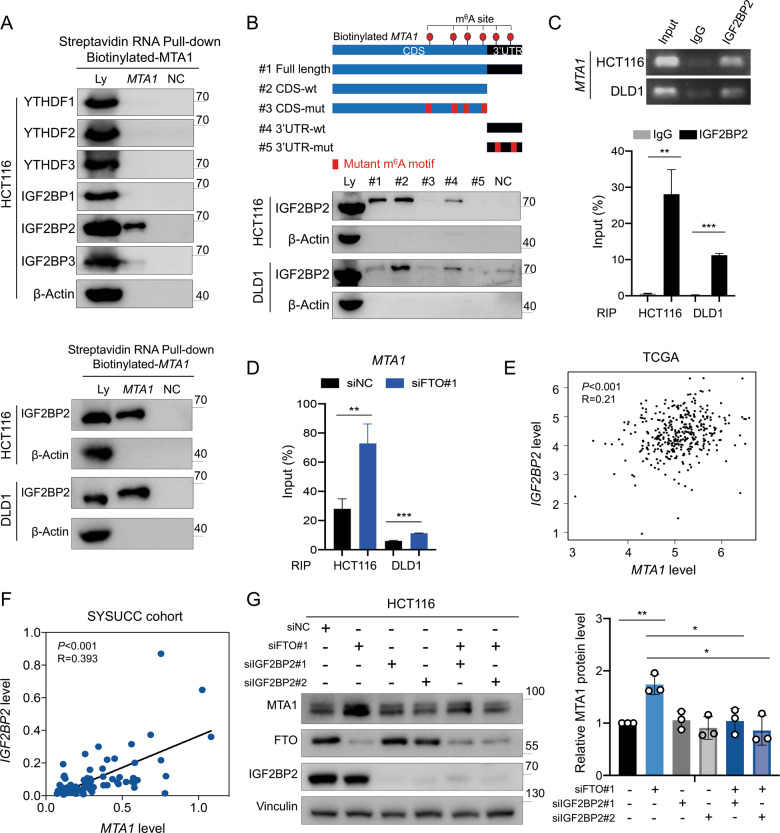
IGF2BP2 specifically binds the *MTA1* transcripts. **A** RNA pulldown was performed with biotinylated *MTA1*. Immunoblotting of YTH family and IGF2BP family m^6^A readers in cell lysate (Ly), biotinylated full-length *MTA1* and beads only (NC) in HCT116 cells (up). Immunoblotting of IGF2BP2 with lysate (Ly), biotinylated full-length *MTA1* and beads only (NC) in HCT116 and DLD1 cells by RNA pulldown assay (down). **B** Immunoblotting of lysate (Ly), *MTA1* full length (#1), *MTA1* CDS region (#2), CDS m^6^A motif mutant *MTA1* (#3), *MTA1* 3’UTR region (#4), 3’UTR m^6^A motif mutant *MTA1* (#5) and beads only (NC) by RNA pulldown assay in HCT116 and DLD1 cells. **C** RIP assays showed the direct binding between IGF2BP2 protein and *MTA1* mRNA in HCT116 and DLD1 cells. Agarose electrophoresis (up) and qPCR analysis (down). **D** RIP assays demonstrating the enrichment of IGF2BP2 protein bound *MTA1* mRNA in siNC versus siFTO HCT116 and DLD1 cells. **E** The correlation between *MTA1* and *IGF2BP2* RNA expression levels in TCGA dataset for COAD and READ assessed by using the GEPIA online tool (http://gepia. cancer-pku.cn/). **F** The correlation between *MTA1* and *IGF2BP2* RNA levels in 78 CRC specimens from SYSUCC. **G** Immunoblotting assay of MTA1, FTO and IGF2BP2 protein levels in HCT116 cells with siNC, FTO knockdown only (siFTO#1), IGF2BP2 knockdown only (siIGF2BP2#1, siIGF2BP2#2) and both FTO and IGF2BP2 knockdown (siFTO#1 + siIGF2BP2#1, siFTO#1 + siIGF2BP2#2). Data in (**C**, **D**, and **G**) are presented as the mean ± SD (*n* = 3). *P < 0.05, **P < 0.01, ***P < 0.001 (Student’s *t* test).

“Writers” and “erasers” determine m^6^A prevalence and distribution, whereas “readers” mediate the downstream effects of the modification. To date, several m^6^A reader proteins have been identified, including members of the YT521-B homology (YTH) family (YTHDF1, YTHDF2, YTHDF3) and the insulin-like growth factor 2 mRNA-binding protein (IGF2BP) family (IGF2BP1, IGF2BP2, IGF2BP3) [11, 28]. RNA pulldown assay was used to identify the specific m^6^A readers of *MTA1* transcripts. We found that IGF2BP2, but not other readers, specifically bound the *MTA1* transcripts in HCT116 cells (Fig. 6A). The direct binding of IGF2BP2 and full-length transcripts of *MTA1* was also verified in both HCT116 and DLD1 cells, and the specific binding was found to be significantly impaired after m^6^A motif mutation (Fig. 6A, B, Supplementary Information: Table S3). In addition, the RIP assay verified the direct binding of IGF2BP2 protein and *MTA1* mRNA in HCT116 and DLD1 cells (Fig. 6C), and the interaction between IGF2BP2 and *MTA1* was enhanced after FTO knockdown (Fig. 6D). In TCGA database, only *IGF2BP2* expression exhibited a positive correlation with *MTA1* expression (Fig. 6E, Supplementary Information: Fig. S6A). Moreover, *IGF2BP2* expression was positively correlated with *MTA1* expression in 78 CRC tumor tissues from the independent SYSUCC cohort (Fig. 6F). The depletion of FTO promoted MTA1 expression, while these effects were attenuated by simultaneous IGF2BP2 knockdown (Figs. 6G, S6B).

### Hypoxia inhibits FTO expression in CRC

**Fig. 7:**
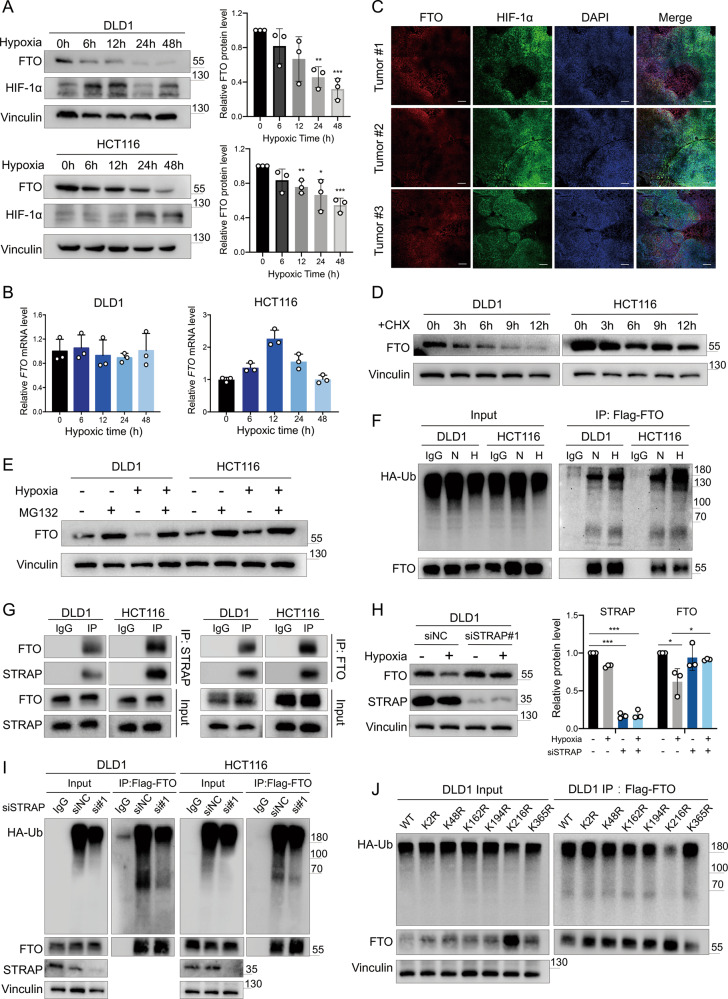
Hypoxia inhibits FTO expression in CRC. **A** Immunoblotting of FTO and HIF-1α levels in DLD1 cells after exposure to hypoxic conditions at several time points (0, 6, 12, 24, 48 h) (left). Relative FTO protein levels (versus Vinculin) (right). **B** Relative *FTO* mRNA level in DLD1 and HCT116 cells after exposure to hypoxic conditions at different time points. **C** Representative fluorescence overlay images of FTO and HIF-1α expression in mouse HCT116 induced subcutaneous tumor tissue. The yellow color in the merged images indicates the colocalization of FTO and HIF-1α. Scale bar: 200 μm **D** Immunoblotting of FTO levels in DLD1 and HCT116 cells followed by treatment with CHX (100 μg/mL) for the indicated times (0, 3, 6, 9, 12 h). **E** Immunoblotting of FTO levels in DLD1 and HCT116 cells cultured under normoxia or hypoxia for 24 h and treated with DMSO or 10 μM MG132 for 12 h. **F** Representative immunoblotting of ubiquitinated FTO from the IP assay of DLD1 and HCT116 cells which were co-transfected with HA-tagged Ub plasmid and Flag-tagged FTO plasmid, then exposed to normoxic (N) and hypoxic (H) conditions for 24 h. **G** The FTO (STRAP) proteins were immunoprecipitated with STRAP (FTO) antibody in DLD1 and HCT116 cells, while IgG pulldown was used for IP control. The immunoblots were probed with FTO and STRAP antibodies to test the interaction between FTO and STRAP. **H** Immunoblotting (left) and relative FTO and STRAP protein levels (right) after STRAP knockdown in DLD1 cells exposed to 21% oxygen and 1% oxygen for 24 h. **I** The immunoblotting of the ubiquitination assay from DLD1 and HCT116 cells after STRAP knockdown and co-transfected with HA-tagged Ub plasmid and Flag-tagged FTO plasmid. **J** Representative immunoblotting of the ubiquitination assay from wild type (WT) or mutant DLD1 cells which were co-transfected with HA-tagged Ub plasmid and Flag-tagged FTO plasmid under hypoxia for 24 h. Data in (**A**, **B**, and **H**) are presented as the means ± S.D. (*n* = 3) *P < 0.05, **P < 0.01, ***P < 0.001. (Student’s *t* test).

As we mentioned above, FTO was downregulated in tumor tissues, with discrepancies between the RNA and protein expression levels. FTO is also known as alpha-ketoglutarate-dependent dioxygenase FTO, and it relies on oxygen to perform its function [29]. Recently, the m^6^A demethylase ALKBH5 was reported to be induced by hypoxia to participate in the regulation of cancer development [10, 30]. Therefore, we wondered whether hypoxia could affect FTO expression. FTO was consistently downregulated under hypoxic conditions, however, intriguingly, this hypoxia-related alteration did not appear at the RNA level (Supplementary Information: Fig. S7A, Fig. 7A, B). In further evaluating the relationship between FTO expression and hypoxia, we found that FTO was decreased in hypoxic regions in vivo (Fig. 7C). We next found that FTO protein expression was not affected by hypoxia-inducible factor 1-alpha (HIF-1α) depletion but was decreased after cycloheximide (CHX) treatment in CRC cells (Figs. 7D, S7B). Then, we treated CRC cells with the proteasomal inhibitor MG132, and the depletion of FTO protein expression induced by hypoxia was rescued by MG132 (Fig. 7E). We also treated CRC cells with autophagy activators (rapamycin) and autophagy inhibitors (3-methyladenine and bafilomycin A1), but there was no effect of autophagy modulation on the expression of FTO under hypoxia (Fig. S7C). Moreover, we found that the FTO ubiquitination level was increased under hypoxia, suggesting that the ubiquitin (Ub)-proteasome pathway mediated FTO protein degradation (Fig. 7F). We identified seven E3 ubiquin ligases of FTO protein by immunocoprecipitationmethod (IP) mass spectrometry (Supplementary Information: Table S4). Then, we used IP assay verified that STRAP directly bound to FTO (Fig. 7G). STRAP inhibition reduced FTO ubiquitination level and abolished hypoxia-induced FTO deficiency (Figs. 7H, I, S7D, E). Together, these results indicated that hypoxia led to lower FTO expression due to the increased ubiquitin-mediated degradation of FTO in CRC. Then we predicted six potential ubiquitination sites of FTO (K2, K48, K162, K194, K216, and K365) by UbiProber [31], and constructed individual lysine to arginine point mutants Flag-tagged FTO plasmids (Supplementary Information: Table S5). The K216R mutation of FTO resulted in reduced ubiquitination of FTO, suggesting the K216 was the major ubiquitination site of FTO under hypoxic condition in CRC cells (Figs. 7J, S7F).

## Discussion

FTO, as an m^6^A demethylase, has been shown to be involved in the occurrence and development of various cancers through different downstream genes. For example, FTO promoted breast cancer progression by increasing the RNA degradation of the pro-apoptosis gene BNIP3 via m^6^A demethylation and a YTHDF2-independent mechanism [6] and promoted glioblastoma tumorigenesis and cancer stem cell self-renewal as a component of the m^6^A regulatory machinery [32]. However, in ovarian cancer, FTO functions as a tumor suppressor by restraining cancer stem cell self-renewal [19]. FTO is also known to inhibit tumorigenesis in hepatocellular carcinoma and intrahepatic cholangiocarcinoma [20, 21]. In addition to these conflicting reports about the role FTO plays in different cancers, the function of FTO in CRC is unclear so far. Our study revealed that FTO exerts a tumor suppressive effect on CRC invasiveness and metastasis, suggesting that FTO might be an important marker for predicting the recurrence and metastasis of CRC. Combined with our previous work on METTL3, these findings lead us to propose a mechanism for m^6^A modification and m^6^A machinery on the tumorigenesis, development and metastasis of CRC.

In this study, the colon cancer cell lines DLD1 and HCT116 were used for phenotypes experiments and downstream gene screening. DLD1 cells are derived from Duke C colon cancer, positive for p53 in the presence of S241F and show strong Wnt signal. HCT116 cells are derived from Duke D colon cancer, with high expression of EMT signatures, and show higher aggressive behavior and higher in-vivo metastasis rates than DLD1 [33, 34]. Our results indicate that in both cell lines, MTA1 is a downstream gene of FTO that can be upregulated by hypoxia-induced FTO ablation via a posttranscriptional regulation mechanism. MTA1 is widely upregulated as a key factor in tumor progression in various types of cancers [35]. It is subject to post-translational modifications as ubiquitination, SUMOylation and acetylation [36–38] and is also reported to be a stress response protein which upregulated under several stress-related conditions, such as heat-shock, ironic radiation, and hypoxia [39].

Methylated RNA requires an m^6^A reader to perform further functions. In our study, IGF2BP2 directly binds to *MTA1* mRNA, and its binding site was consistent with the m^6^A modification site controlled by FTO. Moreover, IGF2BP2 inhibition restrained MTA1 expression in the case of FTO deficiency, suggesting a FTO-IGF2BP2 m^6^A regulatory mechanism of MTA1 expression in CRC. Here, we discovered a new epigenetic mechanism that regulates MTA1 expression and provides a novel therapeutic strategy for targeting MTA1 in CRC. According to previous reports, including work in our group, IGF2BP2 facilitated colorectal cancer progression [13, 40, 41], and our current findings provide another line of evidence supporting its characterization as an oncogene.

Hypoxia is one of the most important characteristics of the tumor microenvironment and it plays an important role in promoting mesenchymal transformation and metastasis of tumor cells [42, 43]. Previous studies suggested that changes in posttranscriptional m^6^A modification was a crucial effector of the hypoxic response [44]. Hypoxia increased ALKBH5 expression through HIF-1α, resulting in the sustaining of target gene expression and eventually enhancing the tumor stemness phenotype [10, 30]. Though the two m^6^A demethylases ALKBH5 and FTO belong to the same AlkB family dioxygenases, it has been found that they exhibited contrary expression tendencies in primary neurons after oxygen deprivation/reoxygenation, and FTO was decreased under hypoxia [45]. However, there is very little understanding of the regulation of tumor hypoxic microenvironment on FTO expression.

Here, we first reported that hypoxia could decrease FTO protein expression but not RNA level in CRC cells. Furthermore, we revealed that the hypoxia-induced FTO depletion was mainly cause by ubiquitin-mediated protein proteasome-associated degradation and STRAP was necessary for FTO degradation as the E3 ligase. Knocking down STRAP could reverse the downregulation of FTO under hypoxia. Previous research showed post-translational ubiquitination on Lys-216 directed FTO protein degradation [46]. Similar results were obtained in our study, we observed that only K216R mutant inhibited FTO ubiquitination under hypoxia condition so we considered that K216 was the major ubiquitination site responsible for hypoxia-induced FTO degradation. These findings indicated a new underlying mechanism of FTO proteostasis regulation and propose a novel m^6^A-dependent gene regulatory mode in the epigenetics of hypoxia-induced cancer metastasis. However, there are limitations in this study. The specific interactions between hypoxia, FTO, and STRAP are not elaborated and the effects of hypoxia tumor microenvironment on cellular m^6^A modification levels mediated by other m^6^A writers, erasers, and readers need further exploration.

## Conclusion

**Fig. 8:**
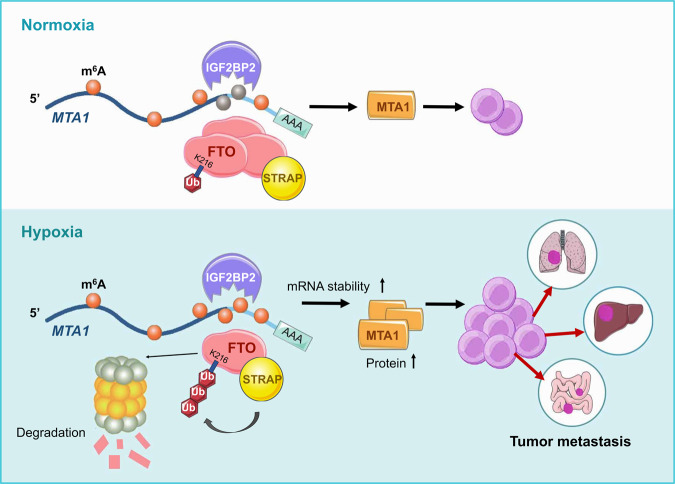
Proposed working model. In CRC cells, hypoxia induces FTO degradation through the ubiquitination-proteasomal pathway. Downregulation of FTO increases *MTA1* mRNA m^6^A methylation, which is recognized by the m^6^A “reader” IGF2BP2, maintaining *\* RNA stability and protein expression, thus leading to acceleration of cancer metastasis and progression.

Our findings identify the tumor suppressive role of FTO in CRC metastasis. MTA1 is the direct target gene of FTO and is regulated by a FTO/IGF2BP2 m^6^A-dependent mechanism. In addition, we found that hypoxia can decrease FTO in CRC cells, mainly by increasing its ubiquitin-mediated protein degradation. We revealed a novel regulatory mode of RNA demethylase FTO degradation exerted by the tumor hypoxic microenvironment, indicating that FTO is a promising predictive factor for CRC metastasis (Fig. 8).

## Materials and methods

### Colorectal cancer patient information and sample collection

Formalin-fixed paraffin-embedded (FFPE) samples from 369 stage I–III CRC patients who underwent radical surgery at Sun Yat-sen University Cancer Center were collected. Of these, paired tumor-adjacent normal tissue specimens were available in 240 cases. The comprehensive clinicopathological information of patients was obtained from medical records. Informed consent was obtained from each participant, and the study was approved by the Institutional Review Board of the Sun Yat-sen University Cancer Center.

### Cell lines and cell culture

The human CRC cell lines HCT8, HCT116, HCT15, DLD1, SW480, SW620, RKO, Ls174t, CW-2, LoVo, and HT-29 were purchased from American Type Culture Collection (Manassas, VA, USA) and Shanghai Cell Bank of Chinese Academy of Sciences (Shanghai, China). The cells were cultured in RPMI 1640 (HyClone, Logan, UT, USA) medium with 10% fetal bovine serum (Invitrogen, Carlsbad, CA, USA) and antibiotics (100 μg/mL penicillin and 100 μg/mL streptomycin) at 37 °C in a 5% CO_2_ cell culture incubator. For hypoxia exposure, cells were cultured under 1% O_2_, 5% CO_2_, and 94% N_2_ using the Anoxomat™ Mark II anaerobic system (Mart Microbiology, Netherlands). All cell lines were authenticated by STR fingerprinting at the Medicine Lab of the Forensic Medicine Department of Sun Yat-sen University (Guangzhou, China) before use.

### Transfection and plasmid construction

SiRNAs for gene expression knockdown were synthesized by RiboBio Co., Ltd. (Guangzhou, China), and the sequences are listed in Supplementary information: Table S6. SiRNAs were transfected using Lipofectamine™ RNAiMAX Transfection Reagent (Invitrogen, 13778030) following the manufacturer’s instructions. Lentiviruses for FTO overexpression and shFTO were synthesized by OBiO Technology (Shanghai) Corp., Ltd. Lentiviruses for MTA1 overexpression were synthesized by Shanghai Genechem Co., Ltd. Cells were infected by lentivirus in the presence of 5 μg/mL polybrene and then selected with 2–4 µg/mL puromycin for one week. The wild type FTO overexpression plasmid, double mutant (R316Q/R322Q and H231A/D233A) FTO plasmid and individual ubiquitination site mutant FLAG-tagged FTO plasmid were constructed by OBiO Technology (Shanghai) Corp., Ltd.

### In vivo tumorigenesis and metastasis models

For the tumorigenesis experiments, 4- to 5-week-old female BALB/c nude mice (Beijing Vital River Laboratory Animal Technology Co., Ltd) were used. Randomization was conducted and mice were treated by an unblinded manner. FTO-overexpressing and control cells (2 × 10^6^ suspended in 100 µL PBS) were subcutaneously injected into each mouse. Tumor size was measured every four days with a caliper, and tumor volume was determined by the standard formula V = ½ (Length × Width^2^). At the end stage, tumor weight was measured.

For the metastasis experiments, two xenograft models were established as described in our previous study [47]. For the orthotopic tumor model, luciferase-labeled FTO overexpressing and control HCT116 cell induced tumors were orthotopically implanted into the cecum. After eight weeks, intestinal mesenteric metastasis and liver metastasis were evaluated by bioluminescent imaging, and the mice were sacrificed. The metastatic nodules in the intestines and livers were counted. For the tail veil injection model, 200 µL of 1 × 10^6^ luciferase-labeled HCT116 cells of different groups were injected into the median tail vein. Distant metastasis was monitored by bioluminescent imaging, and mice were sacrificed at week eight or week ten. Lungs were embedded in paraffin and stained with hematoxylin and eosin (H&E), and the metastatic nodules in the lungs were counted under a microscope. All animal experiments were performed in accordance with protocols approved by our Institutional Animal Care Committee.

### MeRIP qPCR

Total RNA was purified from CRC cells using TRIzol reagent. More than 50 μg of total RNA was used for subsequent RNA fragmentation and immunoprecipitation. The total RNA was fragmented into ~100-nt-long fragments, and 1/10 of the RNA was saved as the input control. PierceTM Protein A/G Magnetic Beads (88803, Thermo Scientific) were washed and resuspended in IP buffer, and 5 μg of anti-m^6^A antibody (202003, Synaptic Systems) or rabbit IgG was added to the beads and incubated by gentle rotation at 4 °C for 2 h. Beads were washed and then mixed with the RNA samples in RIP immunoprecipitation buffer on a rotator at 4 °C for 3 h to overnight. RNA was recovered by the addition of TRIzol after proteinase K digestion, followed by chloroform extraction. Eluted RNAs were precipitated with glycogen and 3 M sodium acetate in 100% ethanol at −80 °C overnight. The immunoprecipitated m^6^A RNAs were reverse transcribed into cDNA and quantified by qPCR according to the procedure described above. Each sample was analyzed with normalization to the input. The primers for MeRIP-qPCR are listed in Supplementary Information: Table S7.

### RNA stability assay

Actinomycin D (ActD), a transcription inhibitor which intercalates into DNA, is widely used for measuring mRNA decay rates [48]. CRC cells of different groups were treated with ActD (S8964, Selleck) at a final concentration of 10 µg/ml. Cells were collected at 0, 3, and 6 h after adding ActD. Total RNA was extracted using TRIzol reagent (15596026, Thermo Fisher Scientific) followed by isopropanol precipitation. *MTA1* mRNA for each group was determined by qRT-PCR assay as described above. Levels of *MTA1* mRNA at each time point were normalized to GAPDH (a highly stable transcript and was independent of FTO activity). Percent mRNA remaining was plotted and determined by nonlinear regression analysis with Prism 8.0 software (GraphPad Software, CA, USA).

### RNA pulldown assays

RNA was transcribed in vitro using the MEGAscript T7 Transcription Kit (AM1334, Thermo Scientific) and labeled with desthiobiotin using the Pierce RNA 3′ End Desthiobiotinylation Kit (20, 163, Thermo Scientific). RNA pulldown assays were carried out with the Pierce Magnetic RNA-Protein Pulldown Kit (20,164, Thermo Scientific) according to the manufacturer’s protocol. Then, 50 pmol of labeled RNA was added to 50 μL prewashed streptavidin beads, mixed gently and incubated at room temperature for 30 min. A 100 μL master mix of RNA-protein binding reaction with 2 mg protein lysate was prepared and added to the RNA-bound beads. After incubation for 30–60 min at 4 °C and washes, the streptavidin beads were heated with 50 μL elution buffer for 5–10 min at 95–100 °C, and the retrieved protein was detected by standard immunoblotting assays.

### RNA immunoprecipitation (RIP) assays

RIP assay was performed by the Magna RIP^TM^ RNA-Binding Protein Immunoprecipitation Kit (#17–700, Millipore Sigma). Briefly, cells were harvested and incubated with magnetic beads which coated with anti-IGF2BP2 antibody (ab#128175, Abcam) or mouse IgG antibody overnight at 4 °C. Then washed the beads six times and incubated with proteinase K to digest the protein. Phenol-chloroform-isoamyl alcohol reagent was used to extract the RNA in the immunoprecipitates and inputs. RT-qPCR was conducted to quantify *MTA1* mRNA. The relative expression was normalized to input and IgG was used to test the specificity of RNA–protein interactions.

### Statistical analysis

Data and bars represent the mean ± standard deviation (SD) of three independent experiments. The sample size determination was account on the need for statistical power. The experiments about patient samples was performed by a blinded manner. A two-tailed Student’s *t* test was used to analyze the differences between groups. Survival curves were constructed for each group using the Kaplan–Meier method and compared statistically by the log rank test. Univariate and multivariate analyses were conducted using Cox regression analysis. Pearson’s chi-square test was used to estimate the correlation between two variables. All analyses were carried out using SPSS (Statistical Package for the Social Sciences) version 18.0 (Chicago, IL, USA) and GraphPad Prism version 8 (GraphPad Software, CA, USA). When the *P* < 0.05, the results were considered to be statistically significant.

For further details, see the online Supplementary Information Methods section. Antibodies used in this study were listed in Supplementary Information: Table S8.

## Supplementary information

Supplementary Information

## Data Availability

All data generated or analyzed during this study are included in this published article and the Supplementary Information files. The RNA-seq and m^6^A-seq data have been deposited in the BioProject database under accession number PRJNA669305.
