# Correction: FTO downregulation mediated by hypoxia facilitates colorectal cancer metastasis

**DOI:** 10.1038/s41388-025-03287-2

**Published:** 2025-02-04

**Authors:** Dan-Yun Ruan, Ting Li, Ying-Nan Wang, Qi Meng, Yang Li, Kai Yu, Min Wang, Jin-Fei Lin, Li-Zhi Luo, De-Shen Wang, Jun-Zhong Lin, Long Bai, Ze-Xian Liu, Qi Zhao, Xiang-Yuan Wu, Huai-Qiang Ju, Rui-Hua Xu

**Affiliations:** 1 State Key Laboratory of Oncology in South China, Collaborative Innovation Center for Cancer Medicine, Sun Yat-sen University Cancer Center, Guangzhou, PR China; 2 Department of Guangdong Key Laboratory of Liver Disease, The Third Affiliated Hospital of Sun Yat-sen University, Guangzhou, PR China; 3 Department of Medical Oncology, Sun Yat-sen University Cancer Center, Guangzhou, PR China; 4 Department of Colorectal Surgery, Sun Yat-sen University Cancer Center, Guangzhou, PR China; 5 Research Unit of Precision Diagnosis and Treatment for Gastrointestinal Cancer, Chinese Academy of Medical Sciences, Guangzhou, PR China

Correction to: *Oncogene* 10.1038/s41388-021-01916-0 published online 03 July 2021

The wrong Supplementary file was originally published with this article; it has now been replaced with the correct file. The original article has been corrected.

## Supplementary information

Supplementary information updated

