# Supplementary material for: Correction: FTO downregulation mediated by hypoxia facilitates colorectal cancer metastasis
Source: Oncogene. 2025 Feb 4;44(8):558. doi: 10.1038/s41388-025-03287-2 (PMC11832410; doi:10.1038/s41388-025-03287-2)
Supplement: Supplementary file 1 — Supplementary information updated [file 41388_2025_3287_MOESM1_ESM.pdf]

Supplementary Information for:

FTO downregulation mediated by hypoxia facilitates colorectal cancer metastasis

Supplementary Methods

Immunohistochemistry (IHC), scoring and immunoblotting

IHC procedures and immunoblotting assays were conducted as previously described[1]. IHC staining intensity was evaluated on a scale of 0 to 3 (0, negative staining; 1, weak staining; 2, moderate staining; 3, strong staining), and the percentage of stained target cells in each category was recorded (0 to 100%). The final IHC score is the sum of each staining intensity multiplied by the corresponding percentage, and the score scale is 0 to 300.

RNA isolation and qPCR analysis

Total RNA was isolated with TRIzol reagent and reverse transcribed to cDNA with the Prime Script RT Master Mix Kit (RR036A, Takara). qRT-PCR assays were performed in a LightCycler 480 instrument (Roche Diagnostics, Switzerland) using GoTaq qPCR Master Mix (A6001, Promega). The relative mRNA levels were calculated with the $2^{-\Delta Ct}$ or $2^{-\Delta\Delta Ct}$ method, and GAPDH was used as an endogenous control. The primers used in this study are listed in Table S7.

Wound healing assays

Marker pen was used to draw horizontal lines on the back of the 6-well plate, then cells were plated to the 6-well plate. When the cells were in an optimal density, used a pipette tip to scratch a wound through the entire center of the well, perpendicular to the marked

horizontal line. Washed the plate with PBS, added serum-free medium and the wound healings were observed at 0, 12, 24 h by a microscope. Image J software was used to calculate the healing area of scratches.

Transwell migration and invasion assays

Transwell migration assays were performed using a 24-well Transwell chamber system (Corning, USA), and invasion assays were performed using a 24-well invasion chamber system (Corning, USA). A total of 1-2 × 10^5 cells in 200 μl of serum-free medium were seeded in the upper chamber, and 600 μl of 20% FBS medium was added to the lower chamber. After 24-36 hours of incubation, cells were fixed with methanol, stained with 0.1% crystal violet, and photographed under a 20× microscope; the numbers of migrated and invaded cells were then counted.

Cell growth and proliferation assays

Cell growth ability was assessed using the MTS assay as previously described[4] and quantified by measuring the absorbance at 490 nm on a SynergyTM Multi-Mode Microplate Reader (Biotek, Vermont, USA).

Transcriptome sequencing

Total RNA was isolated from FTO knockdown and control DLD1 and HCT116 cells using TRIzol reagent and each group had 3 duplicate samples. Sequencing libraries were prepared from 3 μg of total RNA and constructed using the NEBNext® UltraTM Directional RNA Library Prep Kit for Illumina® (NEB, USA). All samples were sequenced on the Illumina HiSeq 4000 platform by Novogene Co., Ltd. Sequence reads were mapped to the human genome version hg19 by using HISAT2 software[2]. The

transcription levels were quantified with the featureCounts tool[3]. Differentially expressed genes (DEGs) between the FTO knockdown and control groups were analyzed by DESeq2. DEGs with log2 > 0 or < 0 and *P* value < 0.05 were defined as upregulated genes or downregulated genes.

Vector and m^{6}A mutation assays

The online tool SRAMP (http://www.cuilab.cn/sramp/) was used to predict potential m^{6}A modification sites on MTA1 RNA sequences. Full-length MTA1 transcripts, the MTA1 CDS region, the MTA1 three prime untranslated region (3′-UTR), and the m^{6}A motif mutant CDS and 3′-UTR regions were cloned into pcDNA3.1 constructs by OBiO Technology (Shanghai) Corp., Ltd. and used for the RNA pulldown assay.

MeRIP sequencing

MeRIP sequencing was performed as previously described with minor modifications[5]. RiboMinusTM Eukaryote Kit v2 (A15020, Invitrogen) was used for RNA isolation and purification. Total RNA (50 μg) was sheared to ~100 nt fragments by RNA Fragmentation Reagents (AM8740, Invitrogen), and 1/10 of the fragment pool was conserved as an input control for further sequencing. The fragmented RNA and anti-m^{6}A antibody (202003, Synaptic Systems) were incubated together for 1 hour at 4°C. PierceTM Protein A/G Magnetic Beads (88803, Thermo Scientific) were washed and resuspended in immunoprecipitation (IP) buffer. Then, the fragmented RNA and m^{6}A antibody mixture were added to the beads for conjugation by gentle rotation at 4°C overnight. The m^{6}A antibody was digested using proteinase K digestion buffer. MeRIP-seq library preparation and sequencing on an Illumina HiSeq 2500 system were carried

out by RiboBio (Guangzhou, China).

MeRIP-seq analysis: The m^{6}A modification peaks were called using the MACS2 algorithm from the input (RNA-seq) and m^{6}A IP (MeRIP-seq) sequencing libraries with a q-value (*P* value with false discovery rate (FDR) correction) of 0.05. The m^{6}A modification peaks that appeared in at least two samples were retained for further analysis. Homer was used for m^{6}A peak annotation, BEDTools was used for m^{6}A read extraction[6], and our custom Perl script was used for conversion to RPKM. The data were normalized using the "limma" package in R software[7].

Immunofluorescence

Frozen sections from mouse subcutaneous tumors were used. Specimens were blocked with 1% bull serum albumin and 0.05% Triton X-100 (all Sigma-Aldrich, USA) for 30 minutes at room temperature. Then, the sections were incubated with primary antibodies (FTO/ab92821, 1:100 diluted; HIF-1A/ab51608, 1:100 diluted) in 1% bull serum albumin at 4°C overnight, followed by fluorophore-conjugated secondary antibodies (Alexa Fluor® 488 anti-rabbit, Alexa Fluor® 647 anti-mouse, both Cell Signaling Technology, USA) for 1 hour in the dark at room temperature. Sections were counterstained with DAPI (ProlongTM Gold Antifade Mountant with DAPI, Thermo Fisher, USA). Images were captured using tile scanning at 20× magnification using a Zeiss LSM 880 Airyscan confocal microscope. Image processing was performed with Zen Blue Software (Carl Zeiss).

Immunoprecipitation (IP) and mass spectrometry

For FTO ubiquitination co-IP analysis, HCT116 and DLD1 cells were co-transfected

with the FLAG-tagged FTO plasmid (OBiO Technology Corp., Ltd, Shanghai, China) and HA-tagged Ub plasmid (Umine bioTechnology Co., Ltd, Shanghai, China). Cells were incubated under hypoxic conditions (1% O_2) and normal conditions (21% O_2) for 24 hours and treated with MG132 for 6 hours. Then, the cells were lysed with IP buffer (Beyotime Biotechnology) and protease inhibitor cocktail (Roche) for 30 minutes on ice and centrifuged at 12,000 × g for 10 minutes at 4°C. The supernatants were incubated with anti-FLAG magnetic beads (MCE) as described in the manufacturer's instructions. Eluted proteins were analyzed by immunoblotting. For the IP assays, anti-FTO antibody and anti-STRAP antibody were used, and the immunoprecipitants were detected by Western blotting.

For mass spectrometry, FLAG-tagged FTO-overexpressing HCT116 cells were lysed as described above, the cell lysates were incubated with anti-FLAG magnetic beads, and eluted proteins were collected and separated by sodium dodecyl sulfate-polyacrylamide gel electrophoresis (SDS-PAGE) and then analyzed by Western blotting. The target band was excised and used for high performance liquid chromatography-mass spectrometer analysis, which was performed by Fitgene Biotech Co., Ltd. The original mass spectrometry files were processed and converted by MM File Conversion software to obtain MGF format files, and protein identification was performed with MASCOT software (http://www.matrixscience.com/) by searching UniProt_Aedis Aegypti.

1. Ju HQ, Lu YX, Chen DL, Tian T, Mo HY, Wei XL, et al. Redox Regulation of Stem-like Cells Though the CD44v-xCT Axis in Colorectal Cancer: Mechanisms and Therapeutic Implications. Theranostics. 2016;6(8):1160-75.
2. Kim D, Langmead B, Salzberg SL. HISAT: a fast spliced aligner with low memory requirements. Nat Methods. 2015;12(4):357-60.
3. Liao Y, Smyth GK, Shi W. featureCounts: an efficient general purpose program for assigning sequence reads to genomic features. Bioinformatics. 2014;30(7):923-30.
4. Ju HQ, Lu YX, Wu QN, Liu J, Zeng ZL, Mo HY, et al. Disrupting G6PD-mediated Redox homeostasis enhances chemosensitivity in colorectal cancer. Oncogene. 2017;36(45):6282-92.
5. Dominissini D, Moshitch-Moshkovitz S, Salmon-Divon M, Amariglio N, Rechavi G. Transcriptome-wide mapping of N(6)-methyladenosine by m(6)A-seq based on immunocapturing and massively parallel sequencing. Nat Protoc. 2013;8(1):176-89.
6. Quinlan AR. BEDTools: The Swiss-Army Tool for Genome Feature Analysis. Curr Protoc Bioinformatics. 2014;47:11 2 1-34.
7. Ritchie ME, Phipson B, Wu D, Hu Y, Law CW, Shi W, et al. limma powers differential expression analyses for RNA-sequencing and microarray studies. Nucleic Acids Res. 2015;43(7):e47.

Supplementary Figures

Figure S1

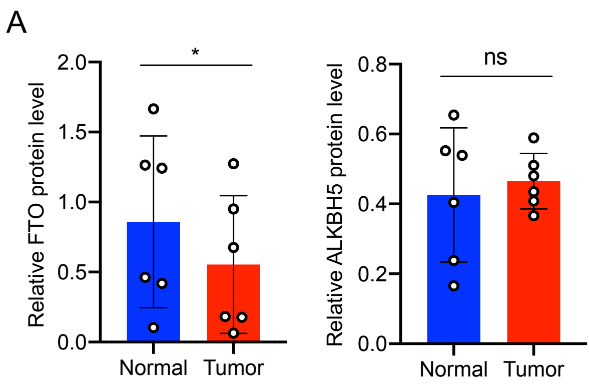

Figure S1. FTO is downregulated in CRC tissues, and its downregulation is associated with poor clinical prognosis.

A. OD quantification ratios for FTO and ALKBH5 protein levels (vs. Vinculin) in 6 paired CRC tumor and adjacent normal tissues, and comparisons were analyzed by two-tailed paired Student's t-test. $^{*}P < 0.05$

Figure S2

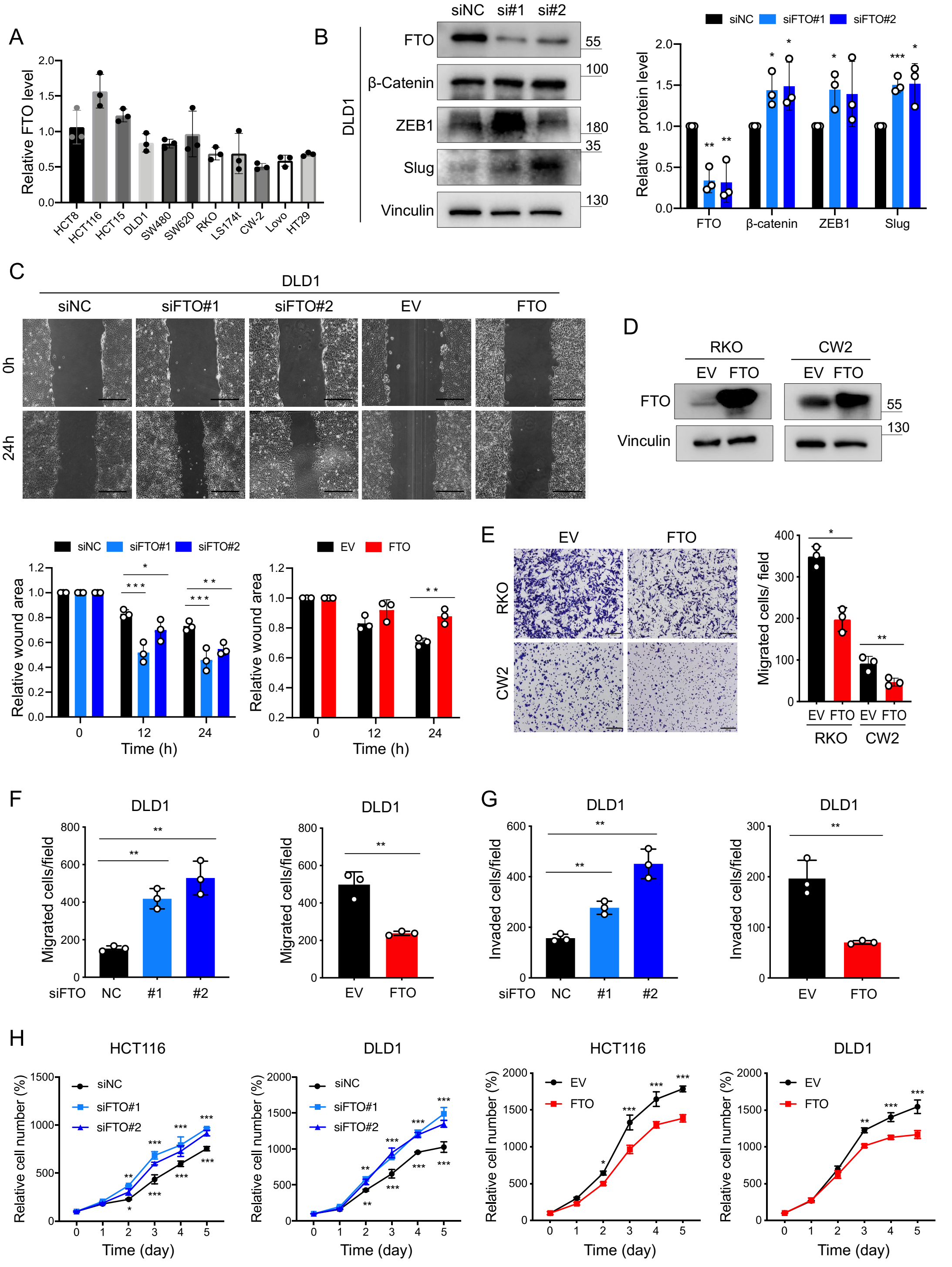

Figure S2. FTO suppresses CRC cell migration and invasion in vitro.

A. Bar graph of FTO protein levels (vs. Vinculin) in 11 CRC cell lines. B. Immunoblotting and bar graph of EMT markers (β-Catenin, ZEB1, Slug) in DLD1 cells after FTO knockdown. C. Wound healing assays of FTO-knockdown (siFTO#1+siFTO#2) versus normal control (siNC) and FTO-overexpressing (FTO) versus empty vector (EV) DLD1 cells were recorded (left) and quantitatively analyzed (right). Scale bar: 400 μm. Two-way ANOVA was used for comparisons at each time point.

D. Immunoblotting of empty vector (EV) versus FTO-overexpressing (FTO) efficiencies in RKO and CW2 cells. E. Images and quantification of transwell migration assays of empty vector (EV) and FTO overexpression (FTO) RKO and CW2 cells. Scale bar: 200 μm. F. Quantification of the transwell migration assays of DLD1 cells. G. Quantification of invasion assays of DLD1 cells. H. MTS assays were performed to determine cell growth after FTO knockdown (siFTO#1, siFTO#2) and in FTO versus EV HCT116 and DLD1 cells, with the absorbance at 490 nm reflecting cell viability. Two-way ANOVA was used for comparisons at each time point in the MTS assays. Data in B, C, E, F and G are presented as the means ± S.D. (n=3) and analyzed by Student's t-test. **P* < 0.05, ***P* < 0.01, ****P* < 0.001

Figure S3

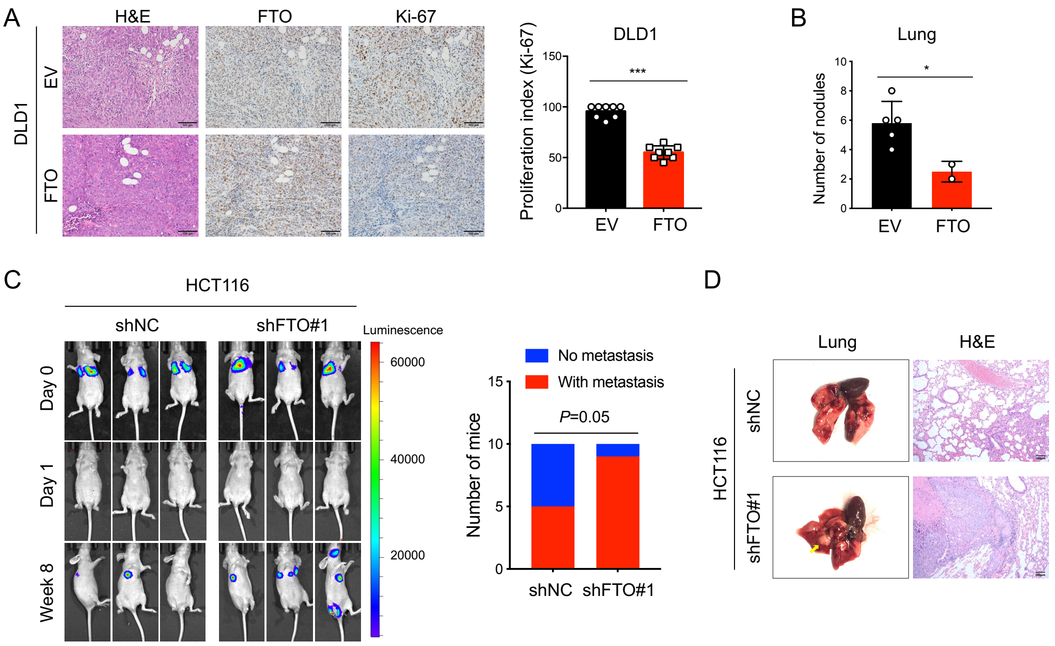

Figure S3. FTO inhibits CRC cell growth and metastasis in vivo.

A. Representative images of hematoxylin and eosin (H&E) staining and IHC staining of FTO and Ki67 antibodies in paraffin-embedded mouse subcutaneous tumor sections derived from the FTO and EV DLD1 cells. Scale bar: 100 μm (left). The Ki67 proliferation index (percent Ki67-positive) in tumor sections from the DLD1 FTO and EV groups was quantified (n=8 mice per group), and two-tailed Student's t-test was used for comparisons (right). B. Comparisons of metastatic lung nodules in EV mice and FTO mice (five sections evaluated per lung), analyzed by Student's t-test. C. The bioluminescent imaging of nude mice tail vein injection metastasis model with shNC and shFTO#1 luciferase-labeled HCT116 cells at days 0, 1 and week 8 (n=10 mice per group). Tumor metastasis formation were followed at week 8 and distant metastasis in shNC and shFTO#1 groups were analyzed by Pearson's chi-square test (right). D. Representative specimen and H&E staining photographs of the metastatic nodules in the lung. **P* < 0.05, ***P* < 0.01, ****P* < 0.001.

Figure S4

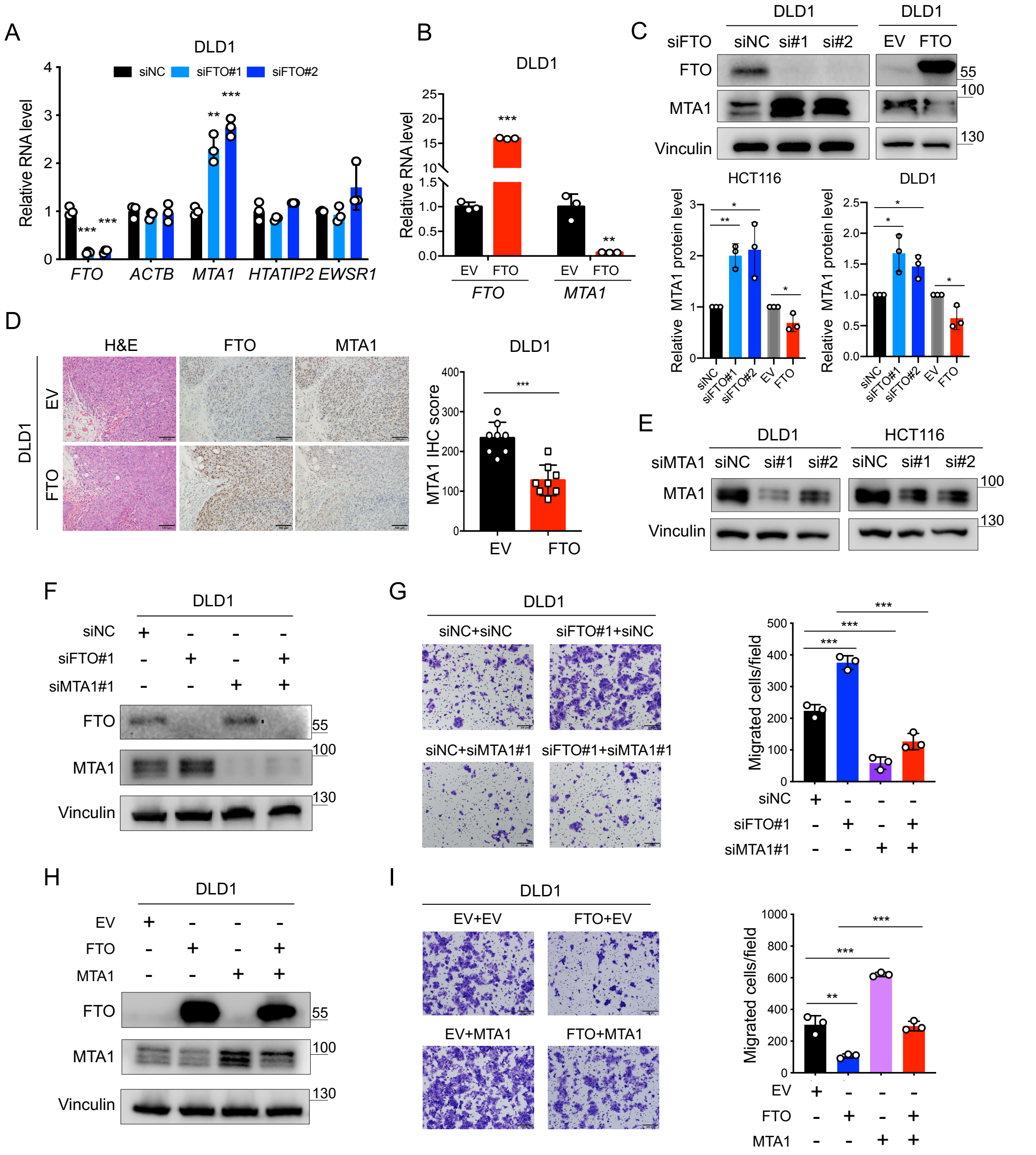

Figure S4. MTA1 is a downstream target gene of FTO.

A. RNA expression levels of the 4 candidate genes after FTO knockdown in DLD1 cells, normalized to GAPDH. B. *MTA1* RNA expression levels were analyzed in FTO and EV DLD1 cells. C. Immunoblotting assay of MTA1 protein levels in DLD1 cells upon FTO knockdown and FTO overexpression (up) and relative MTA1 protein levels (vs. Vinculin) in HCT116 and DLD1 cells (down). D. Representative images of H&E staining and IHC staining of FTO and MTA1 expression in FTO versus EV DLD1 induced tumor tissues (left). Scale bar: 100 μm. IHC scores of MTA1 expression in FTO versus EV groups (n=8 mice per group) (right). E. Immunoblotting of MTA1 knockdown efficiencies in DLD1 and HCT116 cells. F. Immunoblotting assay of FTO and MTA1 protein levels in DLD1 cells with siNC, siFTO#1, siMTA1#1 and siFTO#1+siMTA1#1 (left). G. The images (left) and quantification (right) of migrated DLD1 cells with siNC, siFTO#1+siNC,

siNC+siMTA1#1 and siFTO#1+siMTA1#1. H. Immunoblotting assay of FTO and MTA1 protein levels in DLD1 cells upon EV, FTO overexpression only (FTO+EV), MTA1 overexpression only (EV+MTA1) and both FTO and MTA1 overexpression (FTO+MTA1). I. The images (left) and quantification (right) of migrated DLD1 cells with EV, FTO+EV, EV+MTA1versus FTO+MTA1. Scale bar: 200 μm. Data in A, B, C, G and I are presented as the means ± S.D. (n=3) **P* < 0.05, ***P* < 0.01, ****P* < 0.001. (Student's t-test).

Figure S5

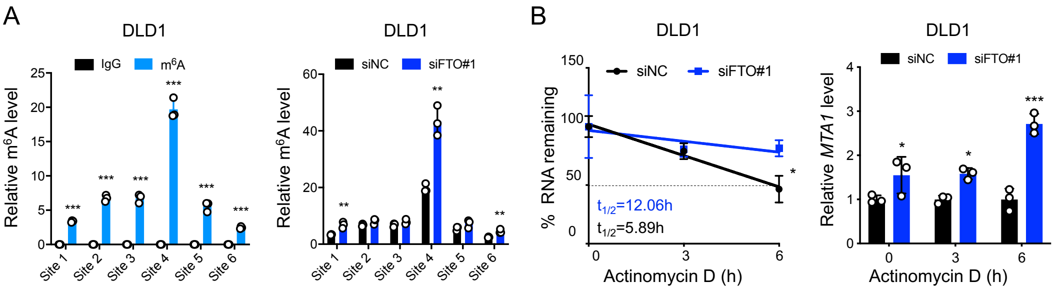

Figure S5. MTA1 is regulated by FTO in an m^{6}A-dependent manner.

A. Enrichment of m^{6}A in different regions of *MTA1* mRNA detected by m^{6}A-RIP qPCR assay in DLD1 cells (left). The m^{6}A methylation of *MTA1* mRNA regions in siFTO#1 versus siNC DLD1 cells by m^{6}A-RIP analysis (right). IgG was used as the negative control. The relative m^{6}A enrichment was normalized by the input. B. The *MTA1* mRNA decay rate was analyzed by nonlinear regression (left), and the relative mRNA expression level of *MTA1* was compared between the two groups at each time point in DLD1 cells(right). Data in A and B are presented as the means ± S.D. **P* < 0.05, ***P* < 0.01, ****P* < 0.001.

Figure S6

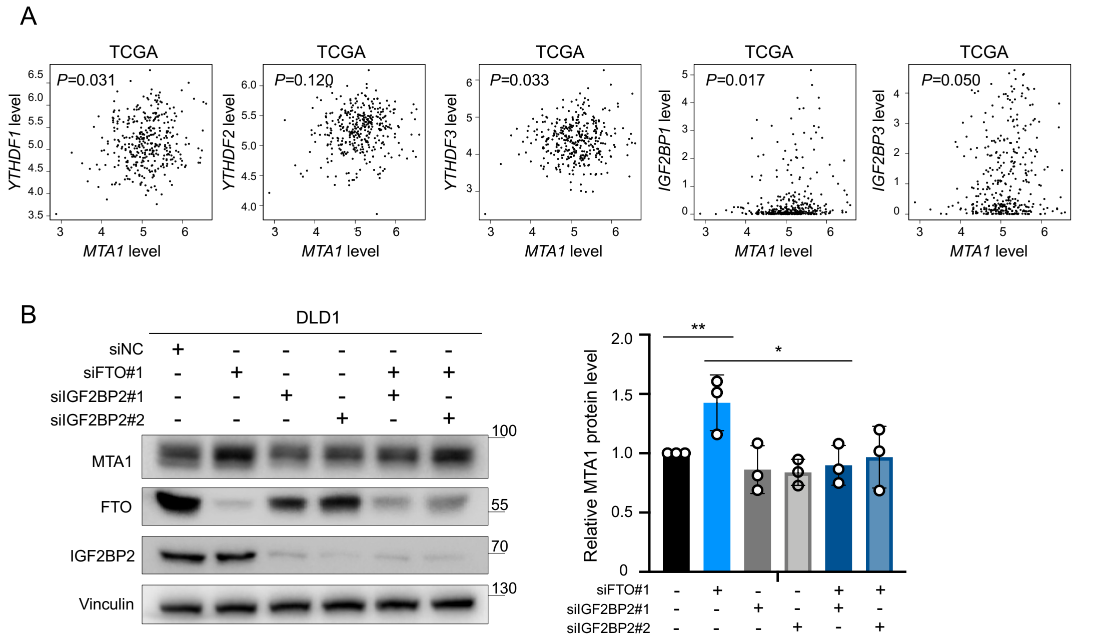

Figure S6. IGF2BP2 specifically binds the *MTA1* transcripts.

A. The correlation between *MTA1* and YTH family (*YTHDF1, YTHDF2, YTHDF3*) m^{6}A readers and other IGF2BP family members (*IGF2BP1, IGF2BP3*) in TCGA dataset for COAD and READ examined by using the GEPIA online tool (http://gepia.cancer-pku.cn/). B. Immunoblotting assay of MTA1, FTO and IGF2BP2 protein levels in DLD1 cells upon siNC, siFTO#1, IGF2BP2 knockdown only (siIGF2BP2#1, siIGF2BP2#2) and both FTO and IGF2BP2 knockdown (siFTO#1+siIGF2BP2#1, siFTO#1+siIGF2BP2#2) (left), and relative MTA1 protein levels (vs. Vinculin) (right). Data in B are presented as the means ± S.D. *$P < 0.05$, **$P < 0.01$, ***$P < 0.001$. (Student's t-test).

Figure S7

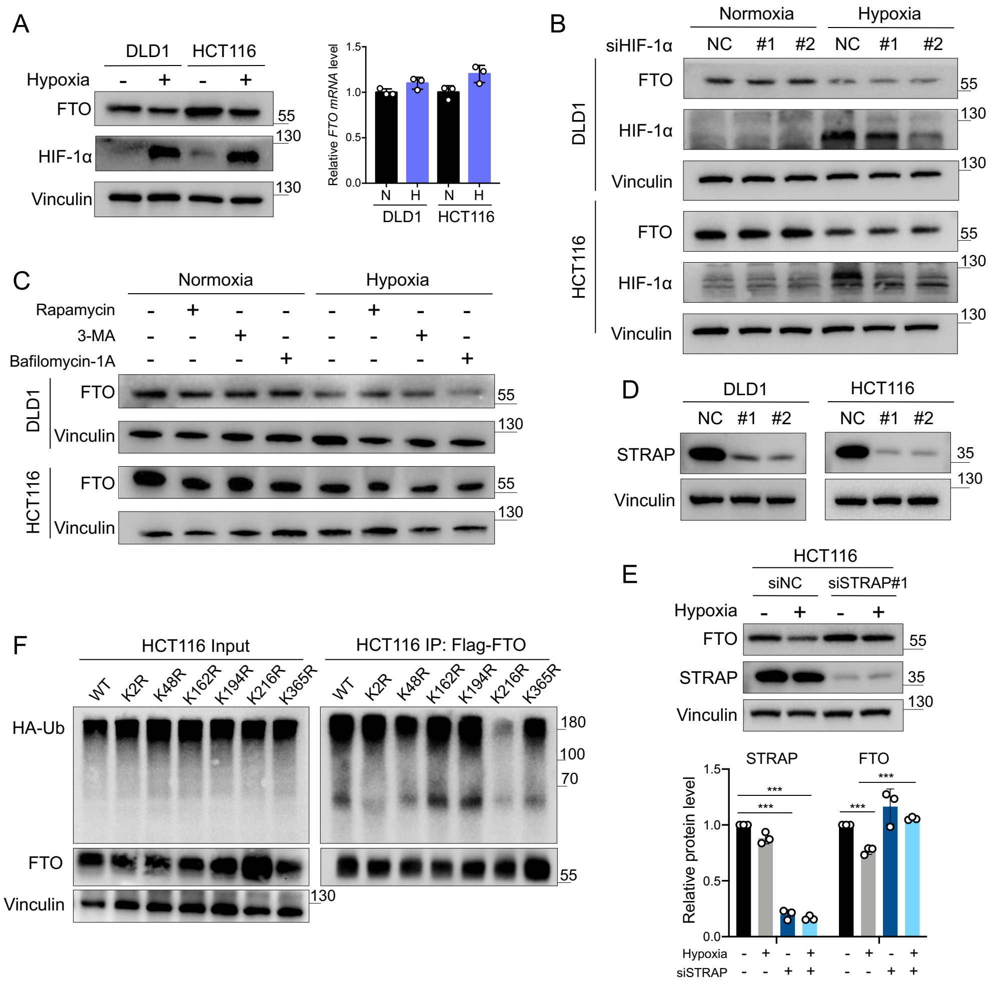

Figure S7. Hypoxia inhibits FTO expression in CRC.

A. FTO and HIF-1α protein expression and FTO RNA level in DLD1 and HCT116 cells after hypoxic induction. Cells were cultured for 24 hours in hypoxia (1.0% oxygen) or normoxia (21% oxygen). B. Immunoblotting of FTO and HIF-1α protein expression in HIF-1α knockdown CRC cells (siHIF-1α#1, siHIF-1α#2) and normal control cells (NC) under normoxic (21% oxygen) or hypoxic (1% oxygen) conditions for 24 hours. C. Immunoblotting of FTO expression in DLD1 and HCT116 cells treated with rapamycin, 3-methyladenine (3-MA), and bafilomycin-1A under normoxia and hypoxia for 24 hours. D. Immunoblotting of STRAP knockdown efficiencies with siRNA#1 and siRNA#2 in DLD1 and HCT116 cells. E. Immunoblotting (up) and relative FTO and STRAP protein levels (down) after STRAP knockdown in HCT116 cells exposed to 21% oxygen and 1% oxygen for 24 hours. F. Representative immunoblotting of the ubiquitination assay FTO from HCT116 cells which were co-transfected with HA-tagged Ub plasmid and Flag-tagged FTO plasmid under hypoxia for 24 hours (either wild type or mutants). Data in A and E are presented as the means ± S.D. (n=3) **P* < 0.05, ***P* < 0.01, ****P* < 0.001. (Student's t-test).

Supplementary Tables

Table S1. Baseline characteristics and correlation analysis for clinicopathologic variables between FTO-high and FTO-low groups of 369 CRC patients

Characteristic	Number (%)	FTO high, n (%)	FTO low, n (%)	*P* value
Age				0.741
>60	177(48)	28(50)	149(47.6)	
≤60	192(52)	28(50)	164(52.4)	
Gender				0.337
Male	209(56.6)	35(62.5)	174(55.6)	
Female	160(43.4)	21(37.5)	139(44.4)	
Location				0.276
Right-sided	103(27.9)	19(33.9)	84(26.8)	
Left-sided	266(72.1)	37(66.1)	229(73.2)	
Degree of differentiation				0.342
Well/moderately	285(77.2)	46(82.1)	239(76.3)	
Poorly	84(22.8)	10(17.9)	74(23.6)	
Nerve invasion				0.202
No	222(60.2)	38(67.9)	184(58.8)	
Yes	147(39.8)	18(32.1)	129(41.2)	
Vascular thrombosis				0.080
No	323(87.5)	53(94.6)	270(86.3)	
Yes	46(12.5)	3(5.4)	43(13.7)	
TNM stage				0.220
I	27(7.3)	5(8.9)	22(7.0)	
II	138(37.4)	26(46.4)	112(35.8)	
III	204(55.3)	25(44.6)	179(57.2)	

Table S2. Univariate and multivariate analyses of factors associated with overall survival (OS) and recurrence-free survival (RFS) of 369 CRC patients

Characteristic, n	Overall survival		Recurrence-free survival	
	Univariate HR, 95% CI, *P*-value	Multivariate HR, 95% CI, *P*-value	Univariate HR, 95% CI, *P*-value	Multivariate HR, 95% CI, *P*-value
Age<br>≤57 (n=192)<br>>57 (n=177)	1.717(1.050-2.806), *P*=0.031	1.778(1.078-2.933) *P*=0.024	1.591 (1.052-2.406) *P*=0.028	1.638 (1.079-2.486) *P*=0.020
Sex<br>Male (n=209)<br>Female (n=160)	1.748 (1.043-2.930), *P*=0.034	1.730(1.026-2.915) *P*=0.040	1.210 (0.799-1.833) *P*=0.368	
Location<br>Left-sided (n=266)<br>Right-sided (n=103)	0.813 (0.464-1.427), *P*=0.471		0.796 (0.493-1.284) *P*=0.350	
Differentiation<br>Well/moderately (n=84)<br>Poorly (n=285)	0.532 (0.319-0.886) *P*=0.015	0.667(0.390-1.142) *P*=0.140	0.635(0.406-0.994) *P*=0.047	0.760 (0.476-1.214) *P*=0.251
Nerve invasion<br>No (n=222)<br>Yes (n=147)	1.950 (1.206-3.153) *P*=0.006	1.504(0.910-2.483) *P*=0.094	1.887 (1.256-2.835) *P*=0.002	1.641 (1.083-2.489) *P*=0.020
Vascular thrombosis<br>No (n=323)<br>Yes (n=46)	2.319 (1.305-4.121) *P*=0.004	1.596(0.868-2.934) *P*=0.132	1.898 (1.135-3.176) *P*=0.015	1.360 (0.790-2.341) *P*=0.267
TNM stage<br>I (n=27)<br>II (n=138)<br>III (n=204)	1.852 (1.184-2.899) *P*=0.007	1.504(0.942-2.401) *P*=0.087	1.692 (1.170-2.446) *P*=0.005	1.401 (0.957-2.052) *P*=0.083
FTO expression<br>Low (n=313)<br>High (n=56)	0.335 (0.122-0.920) *P*=0.034	0.343 (0.125-0.946) *P*=0.039	0.351 (0.153-0.803) *P*=0.013	0.376 (0.164-0.861) *P*=0.021

Table S3. The specific sequences of wild-type or m6A motif mutant *MTA1* CDS and 3’-UTR

Vectors	Sequence*
MTA1-CDS wild-type	AGGATTTCAC**GGACA**TTCAGCAAGA……TGGCAAACCAC**GGACA**GGCCAGGCACATG……GGCGGCGGAT**GAACT**GGATCGACGCCCCG……CGACGAGCCCATCGTCATCGA**GGACT**AG
MTA1-CDS mutation	AGGATTTCAC**GGTCA**TTCAGCAAGA……TGGCAAACCAC**GGTCA**GGCCAGGCACATG……GGCGGCGGAT**GATCT**GGATCGACGCCCCG……CGACGAGCCCATCGTCATCGA**GGTCT**AG
MTA1-3’UTR wild-type	TCCTTGGC**GGACA**CTGGGGGAGGAGAGGAAGAAGCGCGGC**TAACT**TATTCCGAGA……TTATTACTTTTTTTGTAGAT**GAACT**TGAGCTCTGT……
MTA1-3’UTR mutation	TCCTTGGC**GGTCA**CTGGGGGAGGAGAGGAAGAAGCGCGGC**TATCT**TATTCCGAGA……TTATTACTTTTTTTGTAGAT**GATCT**TGAGCTCTGT……
	*The indicated sequence was cloned to the vectors, and the m^{6}A motif sites were bolded and underline.

Table S4. E3 ubiquitin ligases from mass spectrometry

No.	Protein accession	Description	emPAI
1	Q14258|TRI25	E3, E3 activity/RING/RING	0.3
2	Q9BQ67|GRWD1	E3, E3 adaptor/Cullin RING/DCX/DWD	0.21
3	Q13347|EIF3I	E3, E3 adaptor/Cullin RING/DCX/DWD	0.19
4	Q9UMS4|PRP19	E3, E3 activity/RING/U-box	0.19
5	Q9Y3F4|STRAP	E3, E3 adaptor/Cullin RING/DCX/DWD	0.09
6	Q14145|KEAP1	E3, E3 adaptor/Cullin RING/BCR/BTB_3-box	0.09
7	Q9Y6Y0|NS1BP	E3, E3 adaptor/Cullin RING/BCR/BTB_Other	0.09

Table S5. The specific sequences of FTO K/R mutants

ID	Position	Peptide WT	Peptide Mutant
FTO	K2	M**K**RTPTAEERERE	M**R**RTPTAEERERE
FTO	K48	P**K**LILREASSVSEE	P**R**LILREASSVSEE
FTO	K126	E**K**ANEDAVPLCMS	E**R**ANEDAVPLCMS
FTO	K194	I**K**SRAAYNVTLLNF	I**R**SRAAYNVTLLNF
FTO	K216	L**K**EEPYFGMGKMA	L**R**EEPYFGMGKMA
FTO	K365	L**K**QGEEIHNEVEFE	L**R**QGEEIHNEVEFE

Table S6. Sequences of siRNAs used in this study

Name	Si#1	Si#2
FTO	5’-GGATGACTCTCATCTCGAA-3’	5’-GCTGAAATATCCTAAACTA-3’
MTA1	5’-CCAGCATCATTGAGTACTA-3’	5’-GGAAGGATTTCACGGACAT-3’
IGF2BP2	5’-CATGCCGCATGATTCTTGA-3’	5’-GAACGAACTGCAGAACTTA -3’
STRAP	5’-GGATCATGCTACTATGACA-3’	5’-GGGACAGGATAAACTGTTA-3’
HIF1-α	5’-GGAACATGATGGTTCACTT-3’	5’-CTACCCACATACATAAAGA-3’

Table S7. RT-PCR primers used in this study

Name	Forward	Reverse
GAPDH	GGAGCGAGATCCCTCCAAAAT	GGCTGTTGTCATACTTCTCATGG
FTO	GCTGCTTATTTCGGGACCTG	AGCCTGGATTACCAATGAGGA
ALKBH5	ATGCACCCCGGTTGGAAAC	GACTTGCGCCAGTAGTTCTCA
ACTB	TGGATCAGCAAGCAGGAGTA	TCGGCCACATTGTGAACTTT
HTATIP2	CGGAGGGATTTGTTCGTGTTG	AGCTCCTTTAGAGGATAGCAAGT
EWSR1	GGGTATGGCACTGGTGCTTAT	CAGACTGAGCTGCATAGGAGG
MTA1	TTGTCTGTGAGTGGGTTGTGC	TGTT AAAAGAAGGCGAGGAGG
MTA1-site1	AGGGACGAGATGGAGGAGTG	GCACGTATCTGTCGGTGGTC
MTA1-site2	GACGGCAACATGAAGAAGCG	GGGTAGGACTTCCCGTTGAG
MTA1-site3	TGCTCAACGGGAAGTCCTAC	AGAGCAGCTTGCGGATCTTC
MTA1-site4	TGCTCTCATCCTCGGAAACC	TAGTCCTCGATGACGATGGG
MTA1-site5	CTGGGGGAGGAGAGGAAGAA	TCCGCTCCAGCCAAAAAGTA
MTA1-site6	CGGGCCCTAAGGTTTTGTTG	GGCCGCACGATCCTAACATT
STRAP	GCGACCCGTGGTTGATTTG	TGGCGTAGCATAGGTTTACCAT

Table S8. Antibodies for immunoblotting, immunoprecipitation, immunohistochemistry and immunofluorescence in this study.

Primary antibodies	Dilution	Company/Catalog
FTO	WB (1:1000), IHC (1:250), IF (1:100), IP (1:50)	Abcam/ab124892, ab92821 CST#31687
ALKBH5	WB (1:500)	Abcam/ ab69325
Vinculin	WB (1:3000)	Proteintech / 26520-1-AP
β-Catenin	WB (1:1000)	CST#8480
ZEB-1	WB (1:1000)	CST#3396
Slug	WB (1:1000)	CST#9585
β-Actin	WB (1:5000)	Sigma/A5441
MTA1	WB (1:3000), IHC (1:600)	Abcam/ab71153
YTHDF1	WB (1:1000)	Abcam/ab252563
YTHDF2	WB (1:5000)	Proteintech /24744-1-AP
YTHDF3	WB (1:1000)	Abcam/ ab220161
IGF2BP1	WB (1:1000)	Proteintech /22803-1-AP
IGF2BP2	WB (1:1000), IP (20 µg/mg)	Abcam/ab128175
IGF2BP3	WB (1:2000)	Proteintech /14642-1-AP
HIF-1α	WB (1:500)， IF (1:100)	Abcam/ ab51608
anti-HA-Peroxidase	WB (1:1000)	Roche/12013819001
Flag	IP (5ug/sample)	DIA.AN/Cat#: 2064
STRAP	WB (1:1000), IP(4ug/sample)	Proteintech /18277-1-AP
